# The Small GTPase Rac1 Increases Cell Surface Stiffness and Enhances 3D Migration Into Extracellular Matrices

**DOI:** 10.1038/s41598-019-43975-0

**Published:** 2019-05-22

**Authors:** Tom Kunschmann, Stefanie Puder, Tony Fischer, Anika Steffen, Klemens Rottner, Claudia Tanja Mierke

**Affiliations:** 1 University of Leipzig, Faculty of Physics and Earth Science, Peter Debye Institute of Soft Matter Physics, Biological Physics Division, Linnestr. 5, 04103 Leipzig, Germany; 2 Department of Cell Biology, Helmholtz Centre for Infection Research, Inhoffenstr. 7, 38124 Braunschweig, Germany; 3 Division of Molecular Cell Biology, Zoological Institute, Technische Universität Braunschweig, Spielmannstr. 7, 38106 Braunschweig, Germany

**Keywords:** Cellular motility, Biological physics

## Abstract

Membrane ruffling and lamellipodia formation promote the motility of adherent cells in two-dimensional motility assays by mechano-sensing of the microenvironment and initiation of focal adhesions towards their surroundings. Lamellipodium formation is stimulated by small Rho GTPases of the Rac subfamily, since genetic removal of these GTPases abolishes lamellipodium assembly. The relevance of lamellipodial or invadopodial structures for facilitating cellular mechanics and 3D cell motility is still unclear. Here, we hypothesized that Rac1 affects cell mechanics and facilitates 3D invasion. Thus, we explored whether fibroblasts that are genetically deficient for Rac1 (lacking Rac2 and Rac3) harbor altered mechanical properties, such as cellular deformability, intercellular adhesion forces and force exertion, and exhibit alterations in 3D motility. Rac1 knockout and control cells were analyzed for changes in deformability by applying an external force using an optical stretcher. Five Rac1 knockout cell lines were pronouncedly more deformable than Rac1 control cells upon stress application. Using AFM, we found that cell-cell adhesion forces are increased in Rac1 knockout compared to Rac1-expressing fibroblasts. Since mechanical deformability, cell-cell adhesion strength and 3D motility may be functionally connected, we investigated whether increased deformability of Rac1 knockout cells correlates with changes in 3D motility. All five Rac1 knockout clones displayed much lower 3D motility than Rac1-expressing controls. Moreover, force exertion was reduced in Rac1 knockout cells, as assessed by 3D fiber displacement analysis. Interference with cellular stiffness through blocking of actin polymerization by Latrunculin A could not further reduce invasion of Rac1 knockout cells. In contrast, Rac1-expressing controls treated with Latrunculin A were again more deformable and less invasive, suggesting actin polymerization is a major determinant of observed Rac1-dependent effects. Together, we propose that regulation of 3D motility by Rac1 partly involves cellular mechanics such as deformability and exertion of forces.

## Introduction

The migration and invasion of cells are fundamental processes that regulate many relevant functions such as cell positions, fate, their role in morphogenesis, immune responses, regenerative capacities and disease processes such as cancer and deregulation of wound healing events^1–3^. For the translocation of the entire cell body during cell migration on a 2D substrate, cells use a cyclic process compromising the steps of: (i) establishing and maintaining bipolarity (asymmetry), with (ii) extending a leading and establishing an opposing, trailing edge, (iii) adhering to the substratum and (iv) establishing actomyosin-mediated contraction, which promotes the gliding of the cell rear in the direction of migration^1,4–6^. The polarized extension and contraction of cells is regulated by the actin-myosin II interactions, which produce changes in cell shape and facilitate connections to the extracellular tissue microenvironment through cell-matrix adhesion receptors^6–8^.

In a 2D microenvironment, cellular migration is induced by formation of lamellipodia common to various cell types such as fibroblasts, epithelial cells, endothelial cells, cancer cells and different types of leukocytes^9–11^. Lamellipodia consist of an actin filament network, in which the connections between the filaments of the growing actin network are mostly formed through branching mediated by the Arp2/3 complex^12–14^, activated near the plasma membrane at the leading edge of the cell^15–17^. Activation of Arp2/3 complex is driven by Scar/WAVE and associated proteins that build up the so-called WAVE regulatory complex^18–22^.

The WAVE complex is concentrated at the leading edge membrane of lamellipodia^23,24^, and directly activated by the small Rho GTPase Rac1^25,26^. The Rho GTPase family comprises at least 20 members in mammals^27–29^, the best-studied of which comprise the Rac (1, 2, 3) and Rho (A, B, C) subfamilies, regulating lamellipodial actin polymerization and acto-myosin contractility, respectively^5,30,31^. However, it is not entirely clear to what extent Rac inhibition and subsequent suppression of lamellipodia formation^32^ affects cellular motility and migration efficiency in 3D extracellular matrices^33–35^. Additionally, it is unknown how Rac1 affects cellular mechanical properties such as cellular deformability and contractility, both contributing to migration and invasion. Lamellipodia and invadopodia formation are known to promote cellular motility^32,36^. Moreover, in fibroblasts, Rac1 deletion causes complete loss of apparent lamellipodia that can be equally rescued by ectopic expression of any Rac subfamily member (Rac1, 2 and 3), but not by Cdc42, which clearly indicates that expression of at least one Rac subfamily member is essential for the formation of lamellipodia^32^.

In 3D microenvironments, lamellipodia were hitherto not extensively studied, as studies are frequently conducted on stiff 2D substrates or more simple model systems such as fish keratocytes^37^. Similarly, the role of Rac1 during cellular motility was investigated primarily in 2D systems on planar substrates such as wound-healing assays or in transmigration assays through a rigid fixed pore-size membrane using Boyden chambers^32,38^. In rather non-linear elastic 3D matrices, which exhibit a strain-stiffening behavior^39^, lamellipodial-based migration was observed, which coincided with the formation of adhesive or invasive structures called podosomes or invadopodia that occur in hematopoietic or cancer cells, respectively. The latter were particularly involved in tissue invasion and remodeling, including its degradation^40^. In cross-linked and linearly elastic materials such as collagen gels, cells migrate in a high-pressure mode, in which cells exhibit cylindrical, lobopodial structures^39^. However, it is not clear what role Rac1 plays in regulating 3D cellular migration through the extracellular matrix and what type of structures, such as protrusions or membrane blebs, are employed by these cells to facilitate their penetration into 3D microenvironments.

*In vivo* mouse models were used to investigate the function of Rac1 in melanoblasts during neural tube formation in embryogenesis. Rac1 knockout in these cells evoked migration impairments and problems in cell-cycle progression^41^. Moreover, Rac1 activity was also analyzed in normal and disease states of different tissues or during stimulation of a mouse strain expressing a Rac-FRET biosensor. More specifically, Rac activity was found at leading-edge protrusions of neutrophils during migration, and to oscillate during protrusion and stall phases of migration^42^.

The aim of this study was to investigate the precise and functional role of Rac signaling in 3D cell motility, and the impact of Rac GTPases on cellular mechanical properties such as deformability after mechanical stretching of the entire cell. To explore this, we used Rac1 knockout cells (Rac1^−/−^ cells) and corresponding Rac1-expressing control cells (Rac1^fl/fl^ cells). Both cell types were explored on 1.5 g/l fibrillar collagen matrices with subcellularly sized pores serving as artificial 3D extracellular matrix environments, in order to study their invasion capabilities^43,44^. The invasiveness, i.e. the percentage of cells capable of invasion over time and the speed of invasion, depend primarily on mechanical processes including (i) cell adhesion and de-adhesion^45,46^, (ii) cytoskeletal remodeling^43^ and deformability^47^, (iii) protrusive and contractile force generation^45,47^, and (iv) matrix properties such as stiffness, pore size, fibrillar thickness, protein composition and enzymatic degradation^48–50^. Cell invasion strategies (mesenchymal *versus* amoeboid migration) as well as migration/invasion modes (blebbing, protrusive and lobopodial mode) and the speed of migration all depend on the balance of these mechanical parameters^51,52^.

For determining mechanical properties such as deformability, we here used an optical cell stretching device. Indeed, we found that Rac1^−/−^ cells displayed increased deformability and are hence softer than Rac1^fl/fl^ cells. The addition of Rac1-inhibitor EHT1864 also compromised the stiffness of Rac1^fl/fl^ control cells, and rendered the latter more deformable. We also revealed that Rac1^−/−^ cells are less invasive when seeded onto 3D extracellular matrices than Rac1^fl/fl^ cells.

In summary, our data indicate that Rac1 is a key contributor to cell mechanical properties, such as their deformability, which likely affects their capability to migrate into 3D extracellular matrices.

## Results

### Rac1 knockout increases mechanical deformability of cells

**Figure 1:**
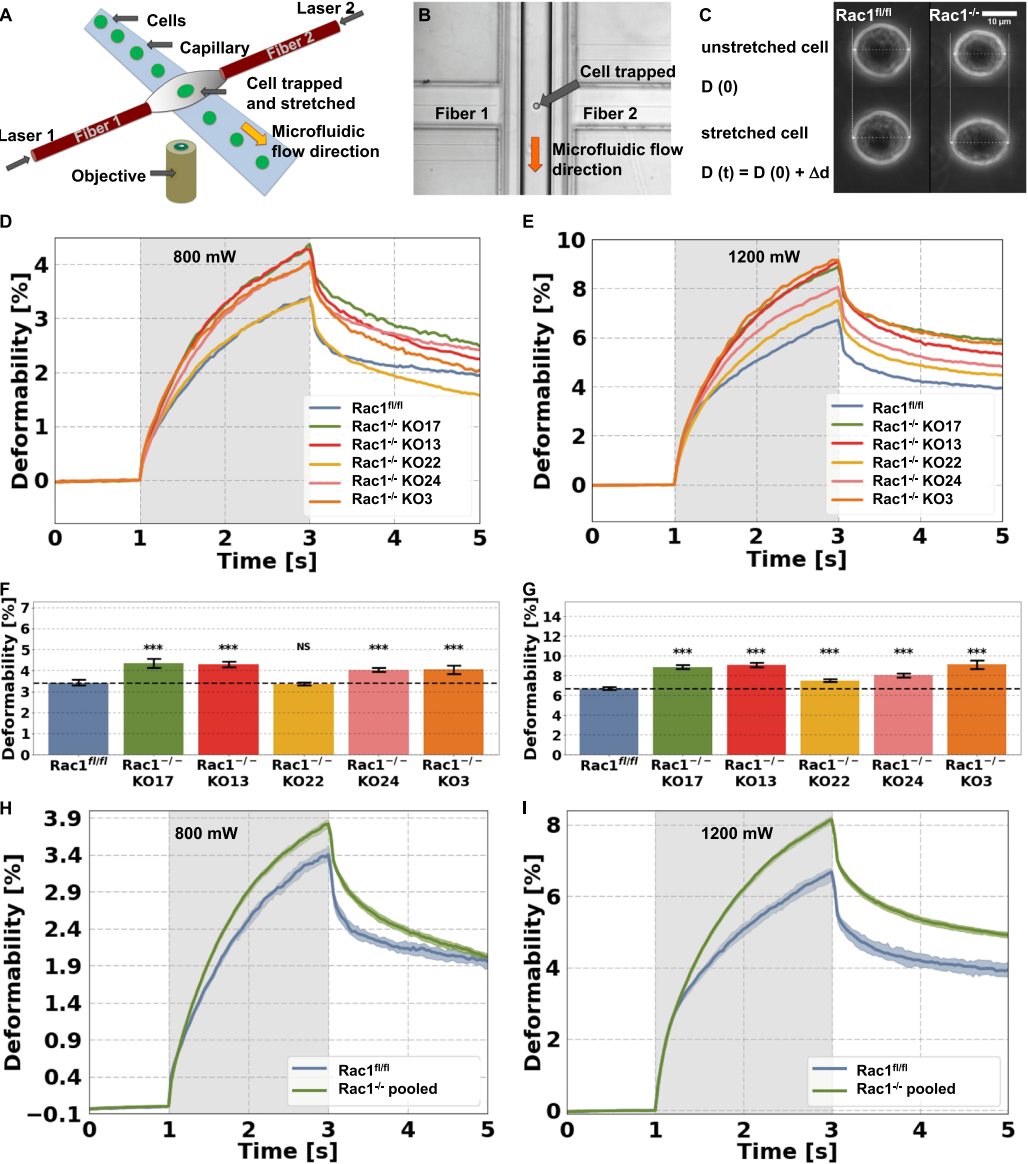
Mechanical properties such as the cellular deformability of five Rac1-deficient (Rac1^−/−^) clones and Rac1^fl/fl^ control cells were measured by using the Optical Cell Stretcher device. (**A**) Schematic image of the optical stretcher setup. (**B**) Image of the optofluidic microchip chamber with a cell that is trapped by the optical force. (**C**) Phase contrast image of an original Rac1^fl/fl^ cell (left images) and an original Rac1^−/−^ cell (right images) trapped (100 mW laser powers) at the onset of the stretching by two opposite laser beams (upper images) and maximally stretched after two seconds of high-power irradiation (1200 mW, lower images). (**D**,**E**) Deformability curves during the first second each cell is trapped, followed by stretching for 2 s with low laser powers of 800 mW (**D**) and high laser powers of 1200 mW (**E**). After the stretching process, the viscoelastic relaxation is observed for 2 s. For low and high stretch forces, Rac1^−/−^ clones KO3, KO13, KO17, KO22, and KO24 were pronouncedly more deformable at their long axis (parallel to the beam axes) compared to Rac1^fl/fl^ cells. (**F**,**G**) Data are presented as median values. Upper bar graphs in F and G are data sets from optical stretcher measurements of all five Rac1^−/−^ cell clones and Rac1^fl/fl^ cells expressed by the creep deformation median values of J (t = 3 s) with SD at 800 mW laser power or 1200 mW laser power, respectively. The deformability of Rac1^−/−^ pooled (all clones are measured together) and Rac1^fl/fl^ cells are shown at a stretch power of 800 mW (**H**) and 1200 mW (**I**). (**F**–**I**) Data are presented as median values with a 95.45% confidence interval. ***p < 0.001and NS = not significant.

We hypothesized that the mechanical properties of cells depend on Rac expression, as this GTPase subfamily plays a role in the structural arrangement of the cytoskeleton underneath the plasma membrane of cells. In order to explore the role of Rac in providing cellular mechanical properties, we investigated the effect of Rac1 gene removal in fibroblasts^32^ (see Fig. S1) on cell mechanical properties such as their deformability. To this end, we used five Rac1 knockout cell clones (Rac1^−/−^) (named KO3, KO13, KO17, KO22 and KO24) that were selected based on relative comparability of growth rates^32^ and their corresponding control (Rac1^fl/fl^) mouse embryonic fibroblast cell line (Fig. 1). In the following, initial optical cell stretching experiments (Fig. 1), we used all five different Rac1^−/−^ cell clones to eliminate clone-specific variations. With a laser-based optical stretching device it is possible to evaluate the entire mechanical properties of living fibroblast cells for both genotypes, i.e. Rac1^−/−^ (note: unless otherwise stated, KO17 was used as representative Rac1 knockout cell line in some follow-up experiments below) and Rac1^fl/fl^ cells. The optical stretcher device constitutes a two-beam laser trap that deforms individual cells in suspension by laser beam-induced surface forces (Fig. 1A–C)^53^. The setup of the optical cell stretcher device can be described as follows: Individual cells are transported to the two opposing laser beams via a microfluidic flow system mounted on a microscopic inverted stage (Fig. 1A). The measurement protocol is described in the following. Individual cells are trapped in the two laser beams with “weak” laser powers of 100 mW (100 mW per laser beam) for one second (Fig. 1B). The trapped cells are stretched by a stepwise increase of the two laser powers from 100 mW (termed trap phase) to “intermediate” laser powers of 800 mW or “high” laser powers of 1200 mW (termed stretch phase). For each cell type, a representative example is shown as a phase contrast image (Fig. 1C). The unstretched (trapped) cells of the two cell types (upper row images) are stretched with laser powers of 1200 mW (lower row images). It is clearly visible that the Rac1^−/−^ cell (clone KO13, right images) is more elongated in the direction of the laser beam (dotted line) compared to the Rac1^fl/fl^ cells (left images).

The deformability of the cell axis parallel to the laser beam axes is monitored over a time interval of 5 s. After trapping the cell for 1 s, the laser powers are raised stepwise from 100 mW to 800 mW (Fig. 1D, grey region) or 1200 mW (Fig. 1E, grey region) for two seconds and then the laser powers are switched back to 100 mW (the cell is still trapped). For another two seconds, the relaxation of the cell from external stress is measured (termed relaxation phase). Due to the broad variability within a non-synchronized cell population, we performed high-throughput measurements and analyzed large numbers for each cell type under individual stretch powers. Thus, we analyzed in total between 496 to 1742 cells for 800 mW stretch laser powers and 411 to 1479 cells for 1200 mW stretch laser powers. For each of the six cell lines, we performed at least five different (independent) experiments (Fig. 1). The measured cell numbers for optical cell stretcher experiments are high, and thus ensure proper and reliable results. The fibroblast cells of both cell types deform over a time period of 2 s and their deformation shows a creep behavior J (t) (Fig. 1D,E). The four Rac1^−/−^ cell clones, KO3, KO13, KO17 and KO24 displayed an increased cellular deformability of their long cell axis (cell axis parallel to laser beam axes) compared to Rac1^fl/fl^ cells (Fig. 1D) for low laser powers of 800 mW employed for cell stretching. In contrast, the fifth Rac1^−/−^ cell clone KO22 behaved similar to Rac1^fl/fl^ cells for low laser powers of 800 mW. However, for the high laser powers of 1200 mW, all five KOs are now more deformable than Rac1^fl/fl^ cells (Fig. 1E), confirming the principle trend of higher deformability in the absence of Rac signaling. The cell size is similar between all six cell lines. Hence, differences in cell deformability cannot be explained by cell size variations (Fig. S2).

Moreover, we determined the median creep deformation at the end of the stretch curve, which represents the maximum deformation. The creep deformation J (t = 3 s) was used to compare the mechanical deformability of the cell lines at low laser powers of 800 mW and high laser powers of 1200 mW (Fig. 1F,G, respectively). Again, these results confirmed that the maximal deformability upon stretch is increased in all clones, except for KO22 at 800 mW, compared to Rac1^fl/fl^ cells (Fig. 1F). At 1200 mW stretch laser powers, however, maximal deformability is increased in all five Rac1^−/−^ cell clones compared to Rac1^fl/fl^ cells (Fig. 1G). In line with this, when pooling all five Rac1^−/−^ cell clones together, the deformability of these Rac1^−/−^ cells is clearly increased as compared to Rac1^fl/fl^ cells at both stretch laser powers (800 mW and 1200 mW, Fig. 1H,I, respectively). Together, we conclude that in spite of some clonal variability, the overall increased deformability of Rac1^−/−^ cells is clearly linked to genotype, since each Rac1-deficient clone is significantly different to Rac1^fl/fl^ cells at least for 1200 mW laser powers. These results thus suggest that Rac1 specifically impacts on deformability, or in other words, positively regulates the inverse, cellular stiffness. The relaxation behavior of Rac1^fl/fl^ and the Rac1^−/−^ cells (all clones) was also significantly different (albeit not completed after 2 s), and hence also Rac1-dependent (Fig. 1D,E,H,I).

### Rac1 knockout decreases cellular invasion into 3D matrices

**Figure 2:**
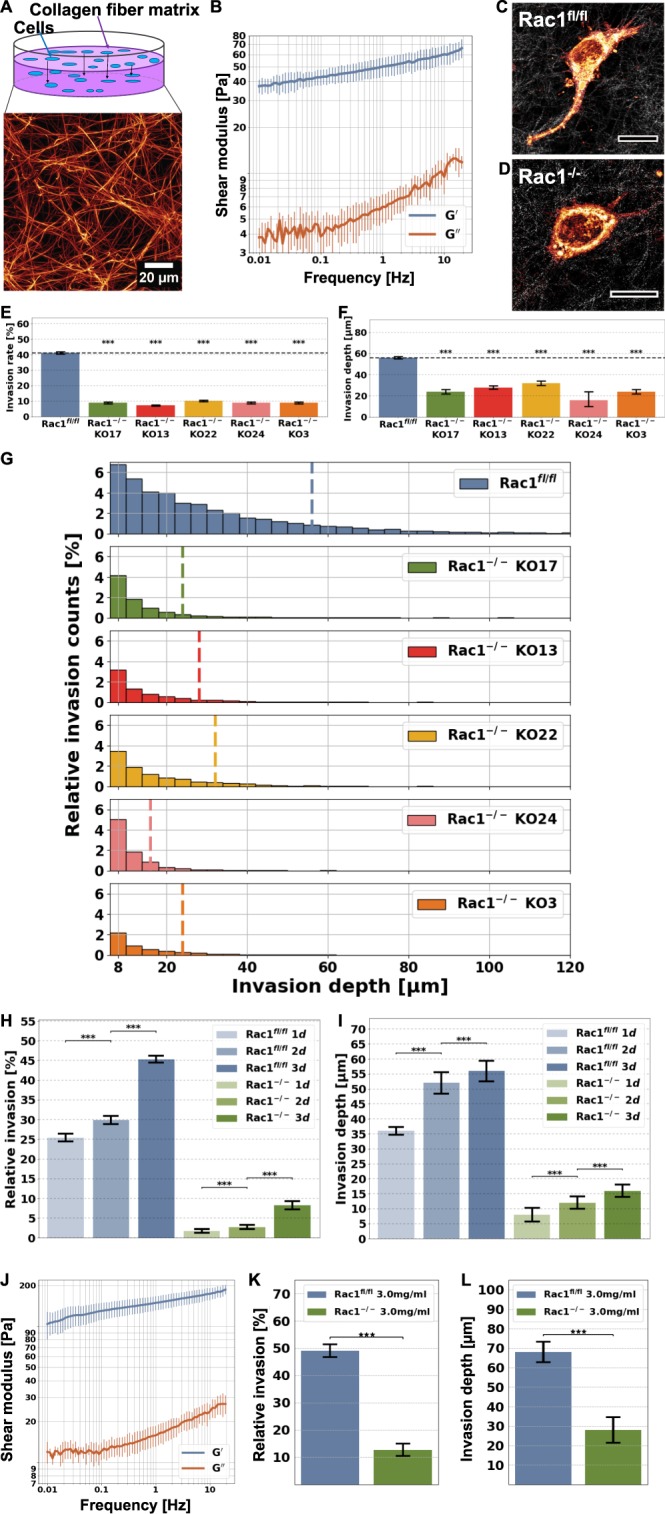
Effect of Rac1 on cellular motility into an engineered 3D microenvironment. (**A**) Experimental setup of the invasion assay, where the cells are seeded on top of the 3D collagen fiber matrix scaffold. (Bottom image) Laser scanning confocal image of a 3D extracellular matrix stained with TAMRA with a collagen concentration of 1.5 mg/ml and a pH of 7.4. Scale bar is 20 µm. (**B**) Plate rheology measurements of 1.5 mg/ml 3D collagen matrices revealed a frequency dependent shear moduli G′ (storage modulus) and G″ (loss modulus). Representative image of an invasive Rac1^fl/fl^ cell (**C**) and an invasive Rac1^−/−^ cell (**D**). Scale bars are 10 µm. (**E**) Average percentage of invasive Rac1^−/−^ cell clones and Rac1^fl/fl^ cells and their invasion depths (**F**) that were migrated in the 3D extracellular matrix after three days of culture at 37 °C, 95% humidity and 5% CO_2_. (**G**) Time course invasion assays: invasion depth profile of the invasive Rac1^fl/fl^ cells (blue) and Rac1^−/−^ cell clones KO17 (green), KO13 (red), KO22 (yellow), KO24 (pink) and KO3 (orange). (**H**) Average percentage of invasive Rac1^−/−^ cell clones (green) and Rac1^fl/fl^ (blue) and their invasion depths (**I**) after 1 d, 2 d and 3 d. (**J**) Plate rheology measurements of 3.0 mg/ml 3D collagen matrices revealed frequency dependent shear moduli G′ and G″. (**K**) Average percentage of invasive Rac1^−/−^ cell clones and Rac1^fl/fl^ cells and their invasion depths (**L**) that migrated into the 3D 3.0 mg/ml collagen matrix after three days of culture. ***p < 0.001and NS = not significant.

Cell mechanical properties such as stiffness and the generation of contractile forces have previously been implicated in driving cell motility and invasion in a one to two mixture of rat and bovine collagen fiber matrices^44,50,54–60^. Moreover, the deformability of cells measured with an optical cell stretcher correlates with their aggressiveness^47,61–63^. We therefore sought to test whether the Rac1-dependent mechanical properties, such as the deformability of cells, determine their motility into 3D extracellular matrix scaffolds, and thus, we seeded Rac1^fl/fl^ cells and each individual clone of the five Rac1^−/−^ cell clones (KO3, KO13, KO17, KO22 and KO24) onto the surface of 3D extracellular matrices harboring a collagen concentration of 1.5 g/l (Fig. 2A). The motility (invasion) assay setup is presented in Fig. 2A. The cells are seeded onto the polymerized 3D collagen fiber matrices with a thickness of roughly 500 µm and cultured for three days in order to allow these cells to migrate into the 3D collagen fiber matrix. A representative laser scanning confocal fluorescent image of the 3D collagen fiber matrix, fluorescently labelled with TAMRA, is shown (Fig. 2A, bottom). We determined the mechanical properties of these collagen matrices using a plate rheometer. The stiffness (shear modulus) of 1.5 mg/ml collagen matrices is around 50.1 ± 7.1 Pa (Fig. 2B), which is comparable to extracellular matrices of specific connective tissues, such as lung or breast tissue^64,65^. Representative example cells of invasive Rac1^fl/fl^ (control) and Rac1^−/−^ cells are presented in Fig. 2C,D, respectively. After three days of cell invasion, we analyzed the percentage of cells that migrated into a 3D extracellular matrix and determined the invasion depths of the cells. For determination of 3D motility, we analyzed a fairly high total number of cells, between 20041 to 51548 cells for each condition (and clone), and performed each experiment at least three times. Since the invasive cells are still healthy, the invasion assays can be performed for at least five days, whereas the optimal time span is from day two to four. Thus, we performed the invasion assay over three days to omit effects of cell death. Indeed, invasiveness was pronouncedly reduced in all five Rac1^−/−^ cell clones compared to Rac1^fl/fl^ cells (Fig. 2E–G). In addition, the invasion profile of the invasive cells shows that each of the five Rac1^−/−^ clones penetrated in smaller numbers and individual cells migrated less deeply into 3D extracellular matrices compared to Rac1^fl/fl^ cells (Fig. 2E–G). In addition to a three-day fixed-time-point invasion assay, we performed a more dynamic invasion assay, in which we determined the percentage of invasion and the invasion depths for Rac1^−/−^ cells (clone KO17) and Rac1^fl/fl^ cells after one, two and three days (Fig. 2H,I). For each time point, the percentage of invasive cells and the invasion depths of Rac1^−/−^ cells were found to be decreased compared to Rac1^fl/fl^ cells. However, the percentage of invasive cells and their invasion depths increased in both cell types over time (Fig. 2H,I), showing that Rac knockout clones were still able to invade, in principle, but much less efficiently. In addition, we investigated the invasiveness of the Rac1^−/−^ cells (clone KO17) and Rac1^fl/fl^ cells into stiffer 3D collagen fiber matrices (Fig. 2J), and found that the invasion rate (Fig. 2K) and invasion depth (Fig. 2L) were increased in both cell types as compared to less stiff matrices, although the pore size of these gels was smaller, with these denser gels therefore representing a higher restriction for cells to migrate into them^50^. These data show that the role of Rac signaling for 3D invasion can vary, dependent on density and stiffness of the 3D matrix employed. Notwithstanding this, our results show that Rac1 functionally affects the motility of fibroblast cells, on the one hand in terms of migration speed (invasion depths), and on the other hand in terms of principal capacity to migrate into dense 3D extracellular matrices (percentage of invasive cells). In order to determine whether the number of cells influenced the percentage of invasive cells (invasion rate), we analyzed whether there is a correlation between relative invasion and cell number, but found no correlation between these two parameters (Fig. S3). Moreover, we also provide representative image stacks of all five KO cell clones, i.e. KO3, KO13, KO17, KO22 and KO24 in comparison to Rac1^fl/fl^ cells, with each of them displaying a clear difference in their invasive capability compared to Rac1^fl/fl^ cells (Fig. S4).

As all Rac1^−/−^ cell clones showed basically a highly similar behavior, we selected one of the Rac1^−/−^ cell clones, clone KO17, for future experiments.

### Actin cytoskeleton and morphology of Rac1^−/−^ and Rac1^fl/fl^ cells are altered depending on the dimensionality of the environment, and their morphological phenotypes are different in 2D and 3D

**Figure 3:**
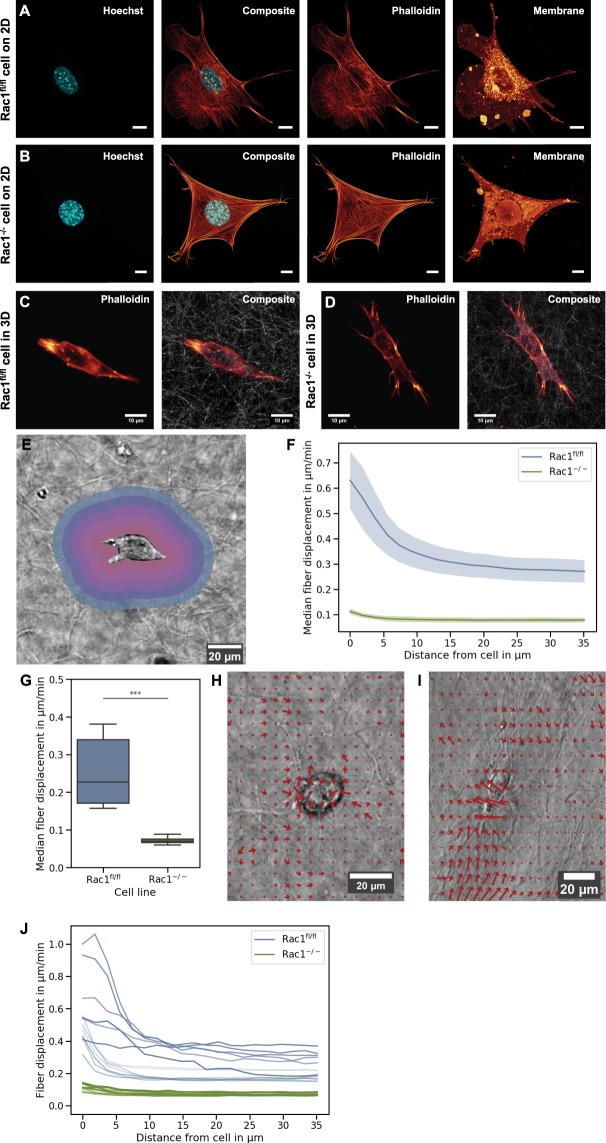
Altered actin cytoskeleton and morphological phenotype of Rac1^−/−^ cells (clone KO17) and Rac1^fl/fl^ cells in 2D compared to 3D microenvironments. Laser scanning confocal images of a representative Rac1^fl/fl^ cell (**A**) or representative Rac1^−/−^ cell (**B**) migrating on 2D planar glass substrates coated with laminin serving as controls for the 3D microenvironments. For both cell types on a 2D substrate, we present for each (from left to right) a 33342 Hoechst staining of the nucleus (blue), a composite image of Alexa Fluor 546 Phalloidin (red) staining of F-actin fibers and Hoechst staining of the nucleus (blue) and a DID staining of the membrane (red). All scale bars are 10 µm. Laser scanning confocal images of a representative Rac1^fl/fl^ cell (**C**) and representative Rac1^−/−^ cell (**D**) that migrated through dense 1.5 mg/ml 3D collagen fiber networks. (**C**,**D**) Fluorescence image of a Rac1^fl/fl^ cell (**C**) or Rac1^−/−^ cell (**D**) stained with Alexa Fluor 546 Phalloidin after migration through a dense 1.5 mg/ml 3D collagen fiber network (left images) and same image combined with reflection mode image of the 3D collagen fiber network (right images). (**E**) Illustration of some sample shells around a representative invasive cell that were analyzed for the determination of the amount of fiber displacement around this cell. (**F**) Increased fiber displacement around invasive cells of Rac1^−/−^ cells (green) compared to invasive Rac1^fl/fl^ cells (blue) at all analyzed distances from cell boundary. (**G**) Median fiber displacement around Rac1^−/−^ cells is reduced to one half compared to that of Rac1^fl/fl^ cells. Values are presented as median with SE. Representative vector fields around a representative invasive Rac1^−/−^ cell (**H**) and Rac1^fl/fl^ cell (I), ***p < 0.001. (**J**) Fiber displacement curves for each individual cell are provided.

We speculated that the differences of the two cell types in cellular deformability or the inverse, stiffness, are based on altered cytoskeletal structures such as microfilaments. Thus, we investigated whether Rac1^−/−^ cells displayed an altered actin cytoskeleton compared to Rac1^fl/fl^ cells. Therefore, we cultured both cell types, Rac1^−/−^ and Rac1^fl/fl^ cells, on planar substrates coated with 10 µg/ml laminin (to ensure proper cell adhesion and control cellular shape integrity)^32^ and in dense 3D extracellular matrices. After 24 hours, cells were fixed and stained with Alexa Fluor 546 phalloidin. Using confocal laser scanning microscopy, the actin cytoskeleton and the cells’ morphology were analyzed by taking z-stacks (with the z-distance between individual images of the stack being approximately 130–200 nm). As reported for planar substrates^32^, Rac1^−/−^ cells clearly lacked lamellipodia and instead exerted other types of cellular protrusions such as filopodia and smaller spike-like structures containing F-actin bundles, while Rac1^fl/fl^ cells displayed lamellipodia (Fig. 3A,B).

In a 3D collagen fiber environment, the Rac1^−/−^ cells displayed more filopodial protrusions at their polarized cell ends, whereas Rac1^fl/fl^ cells were similarly polarized, but displayed a broader (and perhaps stiffer) protrusive structure without exhibiting small, distinct filopodial structures (Fig. 3C,D). These differences in morphology and protrusive behavior may explain the diverse capacities to migrate into and through dense 3D extracellular matrices.

Based on these structural differences observed in the different cell types, we speculated that they may be functionally connected to the alterations in mechanical properties such as cell deformability.

### Rac1^−/−^ cells exert less forces towards their 3D extracellular matrix confinement compared to Rac1^fl/fl^ cells

The cellular mechanical properties, such as the exertion of contracting forces towards confined extracellular matrices, are proposed to contribute to cell invasiveness into 3D matrices. In order to determine the force transmission of the two cell types towards their 3D microenvironment, we analyzed the amount of matrix deformation caused by invasively moving individual cells with Rac1^−/−^ *versus* Rac1^fl/fl^ cells. To this end, we measured the extent of fiber displacement around invaded cells by determining this parameter in “shells” around the cell (Fig. 3E). More specifically, we quantitatively determined the 3D fiber displacement vector field around invading Rac1^−/−^ cells and Rac1^fl/fl^ cells during their migration within the 3D extracellular fiber matrix (Fig. 3F). The median absolute fiber displacements are provided in Fig. 3G. Compared to Rac1^fl/fl^ cells, the absolute fiber displacement levels around Rac1^−/−^ cells were significantly decreased to roughly 29%. While Rac1^−/−^ cells typically altered the collagen fiber network less strongly by distances of 0.072 ± 0.009 µm/min (mean ± SD, n = 14)), Rac1^fl/fl^ cells displaced the collagen fibers more strongly by 0.252 ± 0.087 µm/min (Fig. 3G, n = 13). In addition, a representative vector field is displayed for each cell type, revealing examples for fiber displacements by individual cells for single image planes and at single time-steps (Fig. 3H,I). Fiber displacements by individual cells are also provided (see color code for each cell type, Fig. 3J). In summary, these results demonstrate that Rac1^fl/fl^ cells are capable of deforming their matrix microenvironment more strongly than Rac1^−/−^ cells, indicating that the latter are defective in force transmission, which may explain, at least in part, their inability to efficiently invade into dense 3D extracellular matrices.

### Pharmacological Rac1-inhibition in Rac1^fl/fl^ cells reduces motility in 3D extracellular matrices

**Figure 4:**
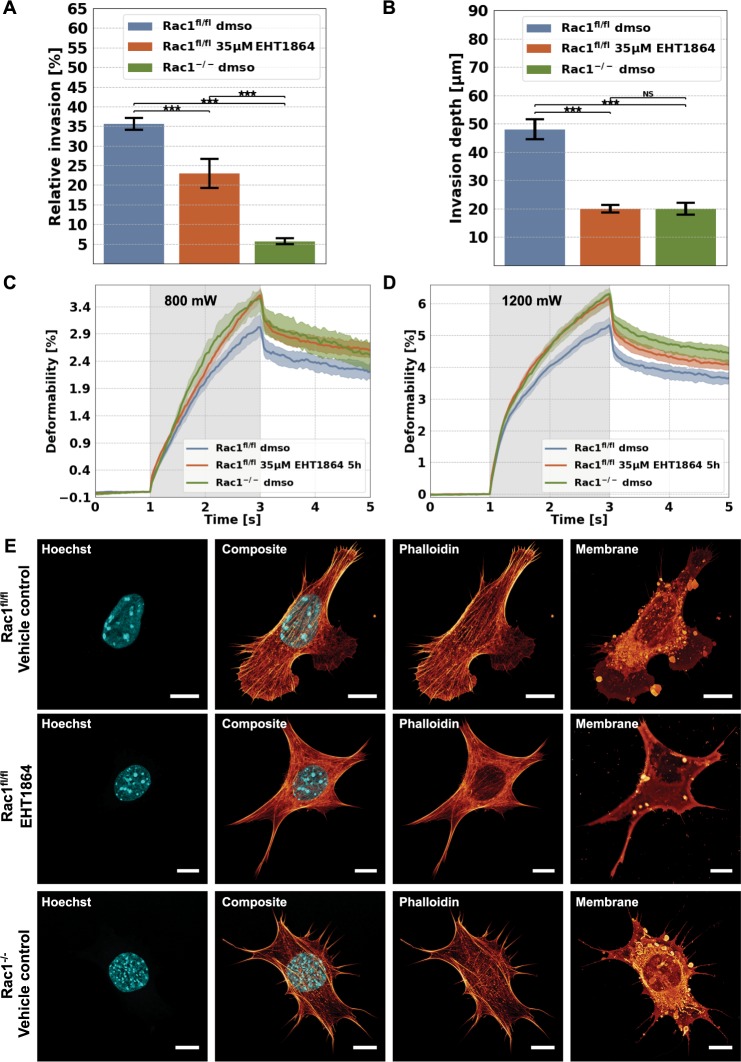
Effect of the Rac1 inhibitor EHT1864 (35 µM) on cellular motility, cellular deformability and the morphology of Rac1^fl/fl^ and for comparison Rac1^−/−^ cells are shown. (**A**) Percentage of invasive cells and (**B**) invasion depths of Rac1^fl/fl^ and Rac1^−/−^ cells. For each condition, between 500 and 1600 cells were analyzed. (**C**,**D**) Long axis deformation of Rac1^fl/fl^ cells (blue) in the presence or absence of treatment with EHT1864 at low “stretch” laser powers of 800 mW (**C**) and high “stretch” laser powers of 1200 mW (**D**), and for comparison the long axis deformation of Rac1^−/−^ cells (green). As control serves each cell type incubated with the DMSO buffer, which has been used as a solvent for the drug. (**E**) Nuclear shape, actin cytoskeletal structure, composite of nuclear shape, actin cytoskeleton and membrane shape of a representative Rac1^fl/fl^ cell in the absence (top row images) or presence of EHT1864 (intermediate row images) and a representative Rac1^−/−^ cell (bottom row images). All scale bars are 10 µm. ***p < 0.001and NS = not significant.

In order to confirm that the differences in 3D motility between Rac1^−/−^ cells and Rac1^fl/fl^ control cells indeed depended on Rac1 expression in the latter, we analyzed in a second approach (shortterm effect) the 3D motility of Rac1^fl/fl^ control cells in the presence of the Rac1 inhibitor EHT1864, used at the comparably moderate concentration of 35 µM. EHT1864 inhibits Rac1 activity by interfering with the effect of the nucleotide binding site and hence interaction of Rac1 with its downstream targets^66^. We also tested higher (up to 100 µM) as well as lower (down to 15 µM) concentrations of EHT1864, and found that 35 µM constituted the best compromise between a pronounced effect and detectable, significant alterations in cell viability, both in 3D collagen invasion assays or in biophysical measurements^67,68^. As expected, the 3D motility of Rac1^fl/fl^ cells was significantly decreased after addition of Rac1 using EHT1864 (Fig. 4A,B). More precisely, the percentage of invasive Rac1^fl/fl^ cells was significantly reduced upon exposure to the inhibitor, and so were their invasion depths (Fig. 4A,B). As vehicle control, we used Rac1^fl/fl^ cells treated with DMSO alone (same concentration as the solvent for EHT1864). Interestingly, Rac1^fl/fl^ in the presence of EHT1864 behaved highly similar to Rac^−/−^ cells treated with DMSO (Fig. 4), except that the latter preformed even worse concerning the percentage of cells being capable of invasion (Fig. 4A), indicating that Rac inhibition was not quite as efficient as genetic removal in this assay. Nevertheless, together with previously shown results, these data suggest that Rac1 functionally contributes to the motility of fibroblasts in dense 3D collagen fiber matrices.

### Inhibition of Rac1 in Rac1^fl/fl^ cells increases cellular deformability

To investigate whether the inhibition of Rac1 by the pharmacological inhibitor EHT1864^67^ leads to altered cellular mechanical properties, we also determined the deformability of Rac1^fl/fl^ cells in the presence of EHT1864 using an optical cell stretcher. We stretched Rac1^fl/fl^ cells along the laser beam axis with low (800 mW) and high laser powers (1200 mW) under the following two conditions. Rac1^fl/fl^ cells were stretched in the presence or absence of EHT1864 Rac1 inhibitor (cells treated with buffer DMSO as a vehicle control) (Fig. 4C,D). For comparison, we included measurements of deformability of Rac1^−/−^ cells, which were also measured in DMSO (to have equal experimental conditions for both cell types). We observed that the Rac1 inhibitor EHT1864 reduces the stiffness of Rac1^fl/fl^ cells significantly, and thus increases the cellular deformability of Rac1^fl/fl^ cells to the level (for 800 mW, Fig. 4C) or nearly the level (for 1200 mW, Fig. 4D) of Rac1^−/−^ cells. Moreover, we proposed that the actin cytoskeleton of EHT1864-treated Rac1^fl/fl^ cells will be altered, as has been reported for Rac1^−/−^ cells that are unable to display lamellipodia^32^. Consistent with these results, we observed that the Rac1 inhibitor EHT1864 impaired lamellipodia formation in Rac1^fl/fl^ cells to an extent similar to Rac1^−/−^ cells. The alterations of the cytoskeleton induced by EHT1864 in Rac1^fl/fl^ cells affirmed the conclusion that Rac1 affects cellular mechanical properties. Moreover, these results also indicated that Rac1 inhibition in Rac1^fl/fl^ cells altered the cytoskeleton in a manner that the deformation of cells was enhanced, highly similar to previous observations with Rac1^−/−^ cells. These findings suggest that the lack of Rac signaling is indeed responsible for the increased deformability and hence, reduced stiffness of Rac1^−/−^ cells observed above. Moreover, since observed again after Rac inhibitor treatment of control cells, this phenotype may also be functionally connected to the impaired motility in dense 3D microenvironments.

### Inhibition of actin polymerization decreases invasiveness of both Rac1^−/−^ and Rac1^fl/fl^ cells

In order to investigate whether the actin cytoskeleton is altered and hence impairs cellular functions in Rac1^−/−^ cells compared to Rac1^fl/fl^ cells, we altered the cell’s architecture by addition of 0.8 µM of the actin polymerization inhibitor Latrunculin A (Lat A). We tested also higher (up to 1.6 µM) and lower (down to 0.2 µM) concentrations and found that 0.8 µM Lat A was the concentration showing a pronounced effect, but without significant alterations in cell viability in 3D collagen invasion assays or biophysical measurements^57,69,70^.

**Figure 5:**
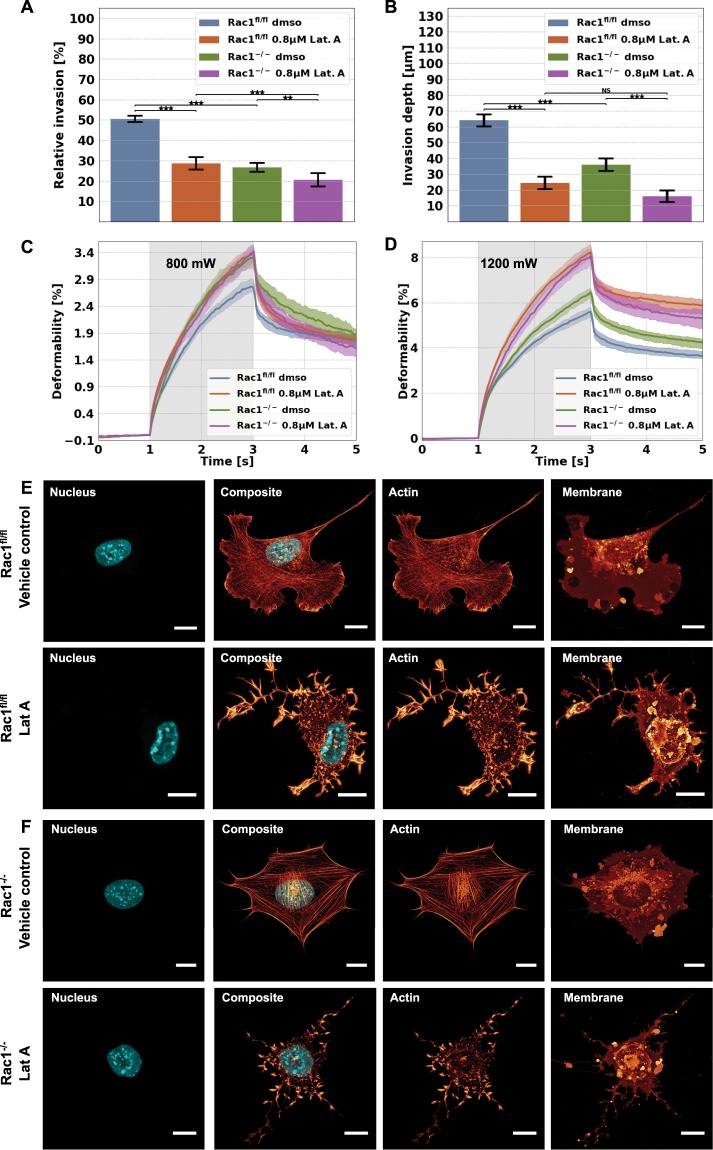
Impact of the actin polymerization inhibitor Lat A (0.8 µM) on cellular motility, cellular deformability and the morphology of Rac1^−/−^ and Rac1^fl/fl^ cells. (**A**) Percentage of invasive cells and (**B**) invasion depths of Rac1^−/−^ and Rac1^fl/fl^ cells. For each condition, between 600 and 1200 cells were analyzed. (**C**,**D**) Long axis deformation of Rac1^fl/fl^ cells (blue) and Rac1^−/−^ cells (green) in the presence of absence of the actin polymerization inhibiting drug Lat A at low stretch laser powers of 800 mW (**C**) and high stretch laser laser powers of 1200 mW (**D**). As control serves each cell type incubated with the DMSO buffer used as solvent for the drug. (**E**,**F**) Nuclear shape, actin cytoskeletal structure, composite of nuclear shape and actin cytoskeleton and membrane shape of a representative Rac1^fl/fl^ cell (**E**) and a representative Rac1^−/−^ cell (**F**) in the absence (top row images) or presence of 8 µM Lat A (bottom row images). All scale bars are 10 µm. ***p < 0.001and NS = not significant.

We analyzed the migration of Rac1^−/−^ and Rac1^fl/fl^ cells in the presence and absence of the actin polymerization inhibitor Lat A at a concentration of 0.8 µM into dense 3D collagen fiber matrices to reveal whether the invasiveness of both cell types can be altered by inhibition of actin polymerization. Lat A is known to inhibit the binding of monomeric G-actin to ATP, and subsequently the polymerization into actin filaments, which we suppose to impact the migration of cells. Consistently, the 3D motility of Rac1^fl/fl^ cells was strongly affected, as reduction of both percentage of invasive cells and their invasion depths were dramatic, but the already quite low performance of Rac1^−/−^ cells was also still reduced by Lat A in a statistically significant fashion, indicating that the remaining activity in the absence of Rac signaling still required active actin polymerization (Fig. 5A,B).

### Inhibition of actin polymerization increases deformability of Rac1^−/−^ and Rac1^fl/fl^ cells

Moreover, we sought to analyze whether the F-actin cytoskeleton is involved in providing cellular mechanical properties such as deformability, and hence we determined the effect of Lat A on Rac1^−/−^ and Rac1^fl/fl^ cells using an optical stretcher with laser powers of 800 mW and 1200 mW (Fig. 5C,D). Not unexpectedly, we detected that the cellular deformability of Rac1^−/−^ cells is not altered with low stretch laser powers of 800 mW, whereas the deformability of Rac1^fl/fl^ cells is significantly increased after Lat A treatment, similar to the levels of Rac1^−/−^ cells without Lat A treatment (Fig. 5C). At higher stretch laser powers, both Rac1^−/−^ and Rac1^fl/fl^ cells exhibited significantly increased deformability, indicating that the depolymerization of the actin cytoskeleton is responsible for the increased deformability of these cells (Fig. 5D).

Moreover, we proposed that the actin cytoskeleton of Lat A-treated Rac1^fl/fl^ cells and Rac1^−/−^ cells is altered. Indeed, we additionally observed that Lat A suppressed filopodia formation in Rac1^−/−^ cells and lamellipodia formation in Rac1^fl/fl^ cells using confocal laser scanning microscopy. Moreover, after treatment with Lat A, F-actin stress fibers were no longer detectable in both cell types (Fig. 5E,F). Finally, these vast alterations of the cytoskeleton induced by Lat A treatments appeared similarly dramatic in both Rac1^−/−^ and Rac1^fl/fl^ cells.

### Cell-cell adhesion forces are increased in Rac1^−/−^ cells compared to Rac1^fl/fl^ cells

**Figure 6:**
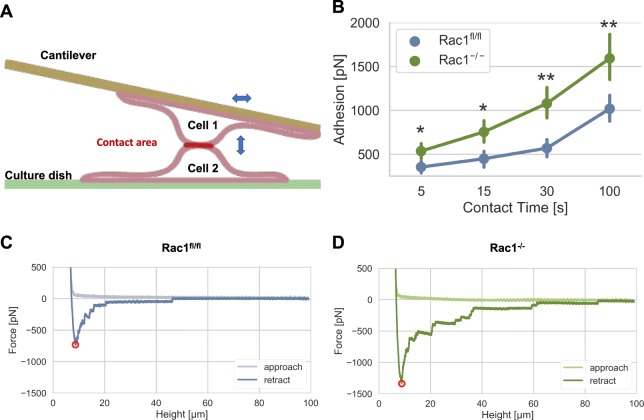
Rac1 knockout impacts on cell-cell adhesion strength using atomic force microscopy (AFM). (**A**) Schematic setup of the AFM measurement with cantilevers carrying a cell, which is pressed with 0.5 nN onto a single adherent cell of the same cell type. (**B**) Cell-cell adhesion forces between two cells of the same cell type of Rac1^−/−^ and Rac1^fl/fl^ cells. For each contact time and cell type, between 8–25 cells were analyzed. Representative force-height curves at 100 s cell-cell adhesion time of Rac1^fl/fl^ control cells (**C**) and Rac1^−/−^ cells are presented (**D**). *p < 0.05 and **p < 0.01.

Rac1 has been found to interact with intercellular cell-cell adherence junctions and to play a role in the stabilization of cell-cell contacts. Thus, we assumed that knockout of Rac1 in cells can alter its cell-cell adhesion forces compared to Rac1^fl/fl^ cells. Moreover, we hypothesized a potential relationship between cell-cell adhesion forces and invasion, since Rac1^−/−^ cells show increased collective invasion compared to Rac1^fl/fl^ cells (Fig. S5). Indeed, such collective invasion phenomena could be explained by increased cell-cell adhesion forces, which may be provided by altered expression of cell-cell adhesion molecules or increased activity of cell-cell adhesion receptors. Thus, the distinct migration capacities of the two cell types might in fact coincide with altered cell-cell adhesion forces developed in Rac1^−/−^ cells. To investigate whether Rac1^−/−^ cells indeed showed altered intercellular adhesion forces compared to Rac1^fl/fl^ cells, we performed cell-cell adhesion force measurements, using a variant of atomic force microscopy, also termed single cell force spectroscopy (Fig. 6A). For this, we attached a cell to the lower side of a cantilever. This is then pressed with 0.5 nN (low force) onto a single adherent cell in a cell culture dish of the same cell type for different adhesion time points, i.e. 5 s, 15 s, 30 s and 100 s. After each adhesion time (which represents a different measurement) the cantilever is retracted (Fig. 6B). During the interaction of the two cells, the indentation depth was kept constant during all measured contact times and in both cell types, whose cell nuclei are of comparable size, so that the contact area could be easily kept constant (Fig. S6). During retraction of the cantilever, we determined the force required to tear both cells apart. Rac1^−/−^ cells displayed significantly higher cell-cell adhesion forces compared to Rac1^fl/fl^ cells for all cell-cell adhesion times. After 100 s, the cell-cell adhesion forces are highest in both cell types and the differences in intercellular cell adhesion strength are clearly visible, as seen in the representative measurement curves of Rac1^fl/fl^ and Rac1^−/−^ cells (Fig. 6C,D, respectively). These findings suggest that Rac1 removal facilitates enhanced cell-cell interactions.

## Discussion

Cell motility is a sophisticated complex process and relies on mechanical and biochemical properties of the cell and the mechanical properties of their microenvironment. Since it has been hypothesized that protrusive lamellipodial structures may be present in a 3D microenvironment^71^, we investigated whether the migratory capacity of the cells is regulated by Rac1-driven lamellipodia formation. Here, we demonstrated that Rac-deficient fibroblast cells (Rac1^−/−^) show decreased invasiveness into 3D extracellular matrices as compared to their parental control cells (Rac1^fl/fl^). In addition, we found that all five individual Rac1^−/−^ cell clones exhibited the same migration behavior in these artificial 3D collagen fiber matrices. These results are consistent with the 2D wound healing migration assays performed on flat substrates^32^. In these 2D motility assays, the Rac1^−/−^ cells could not form lamellipodia and were therefore less motile compared to lamellipodia-forming Rac1^fl/fl^ cells, which migrated in a protrusive, lamellipodia-driven migration mode^32^. When analyzing the cellular morphology in 3D extracellular matrices, we found that lamellipodial protrusive structures were absent in Rac1^−/−^ cells that migrated in a 3D microenvironment; instead, they displayed multiple filopodial, protrusive structures, whereas Rac1^fl/fl^ cells formed small, lamellipodia-like, protrusive structures.

We focused in this study on the mechanism that reduces the invasiveness of Rac1^−/−^ cells. The mechanisms promoting cell motility or cell invasion are still incompletely investigated, however, mechanical factors have been suggested to determine the speed and efficiency of cell migration in dense 3D extracellular matrices^43,45,48,56,57,69,72,73^. These factors include adhesion forces, degradation of the extracellular matrix through secretion of matrix-degrading enzymes, cytoskeletal dynamics, cellular deformability (stiffness) and fluidity, and contractile force generation and transmission^43,47,48,50,51^.

To investigate which of these mechanical factors may contribute to the Rac1-dependent invasiveness of fibroblast cells, we analyzed cellular deformability of different Rac1^−/−^ cell clones (KO3, KO13, KO17, KO22 and KO24) as compared to their parental control cell line (Rac1^fl/fl^). We used an optical cell stretcher, with the help of which we can probe mechanical deformability of cells independently of cell adhesion. For each of those Rac1^−/−^ cell lines, we found that the cells displayed increased deformability compared to Rac1^fl/fl^ cells. This finding is consistent for all Rac1^−/−^ cell lines at 800 mW and 1200 mW laser powers employed for deformation of a cell along its long axis (parallel to the laser beam), except for a single clone, KO22, for which the cells stretched at 800 mW behaved similarly to Rac1^fl/fl^ cells. At 1200 mW laser stretch powers, however, all five Rac1 knockout cell lines (including KO22) were more deformable than Rac1^fl/fl^ cells. Moreover, when we pooled all data for the five clones, the differences in deformability between Rac1^−/−^ and Rac1^fl/fl^ cells were significantly pronounced for both stretch laser powers. We not only found an increased cellular deformability in Rac1^−/−^ cell clones compared to Rac1^fl/fl^ cells, but Rac removal also caused severely decreased invasion. Our results thus contrast with several other studies reporting that the invasiveness of specific cancer cells is increased when the deformability is increased^47,50,62,63,74^. However, there are other studies that agree with our results, since they show increased deformability (decreased stiffness) and decreased invasiveness of distinct cancer cells and fibroblasts^43,56,57,69^. It is worth asking whether these apparently contradictory findings may be explained by the use of distinct methodology, in particular since softness or stiffness of cancer cells has been determined with either optical cell stretcher (adhesion-independent)^47,62,63,74^, magnetic tweezer (adhesion-dependent)^43,56,57,73^ or AFM (both adhesion-dependent and -independent)^50^. However, cancer cells frequently appeared to display increased invasiveness if they were softer, in a fashion independent of how softness was measured, i.e. with optical cell stretcher^47,62,63,74^ or AFM, at least when used on suspended cells^50^. In contrast, if AFM measurements were performed with adherent cancer cells, differences in softness between differentially invasive tumor cell lines were abolished^50^. Furthermore, investigating subcell lines of the human breast cancer cell line MDA-MB-231, the stiffer subcell lines (analyzed with magnetic tweezer) also displayed increased invasiveness^43,57^. All these data suggest that the choice of conditions in which cells are assessed for their cortical stiffness plays a more significant role than the methodology employed. However, since measurements with conditions and methodology identical to those used here have apparently revealed a negative correlation between invasive capacity and stiffness in cancer cells^47,62,63,74^, we conclude that these differences in observations may be caused by the differential behavior of different cell types used. This view would also be consistent with the previously described, positive correlation between stiffness and invasive potential in another type of fibroblast, independently generated from the ones used in the current study^69^. It is also worth noting in this context that the described negative correlation between deformability and invasive potential cannot necessarily be generalized for all tumor types^75,76^. Hence, general differences may exist in cells concerning their mechanical properties and their relation to invasion capacity.

An effect similar to interference with Rac signaling was also observed upon addition of a Rac1 inhibitor to MDA-MB-231 human breast cancer cells, in which the invasiveness of Rac1 inhibitor-treated cancer cells was pronouncedly reduced after three days^54^. Indeed, we could confirm these results, as we performed 3D motility assays in the presence and absence of the Rac1 inhibitor EHT1864 using Rac1^fl/fl^ fibroblast cells. In more detail, we demonstrated that the motility such as the percentage of invasive cells and their invasion depth were reduced in Rac1 inhibitor-treated Rac1^fl/fl^ cells. However, the percentage of invasive Rac1^fl/fl^ cells treated with the Rac1 inhibitor was significantly lower compared to Rac1^fl/fl^ control cells, and still higher than Rac1^−/−^ cells. Hence, the inhibitor confirms the effects observed upon genetic Rac1^−/−^ removal, but it is less effective perhaps than the Rac1 knockout with respect to impairment of cell invasion, indicating that solely genetic removal can lead to the strongest effects on cellular invasiveness. Concerning the mechanical properties such as cellular deformability, the addition of EHT1864 to Rac1^fl/fl^ cells increased their deformability to levels similar to Rac1^−/−^ cells, indicating that cellular deformability indeed does require the inactivation of Rac1. These results suggest that the inhibition of Rac1 activity using EHT1864 is sufficient to reach a similar deformability increase, as has been observed with the Rac1^−/−^ cells for both, 800 and 1200 mW, laser stretch powers.

The recovery of Rac1^−/−^ and Rac1^fl/fl^ cells after deformation is different and may be based on different active and passive parts (or processes) that are involved in this relaxation behavior. Moreover, it can be hypothesized that the deformation of Rac1^−/−^ cells caused plastic deformation and hence longer relaxation times, which need to be measured more precisely in future optical cell stretching experiments.

When the cells are extended to a maximum of 10% of their original length, the deformations are rather small. It hence can be suggested that the membrane-cortical layer interface and the cytoplasmic network of the semi-flexible polymer actin are major contributors to the mechanical properties of cells^47,77,78^. We hypothesized that the increased deformability of Rac1^−/−^ cells may be based on a more fluidized actin cytoskeleton. The elevated actin fluidization may be based on increased actin filament depolymerization, which has been reported for isolated actin filaments^79^ or relies on enhanced acto-myosin interactions such as the increased actin filament sliding, as has been described for isolated actin filaments and myosin interactions^80^. When we further increased the fluidization of actin by inducing actin filament depolymerization through the addition of Lat A, we found that the depolymerization of actin increased the deformability of Rac1^fl/fl^ cells to levels comparable to Rac1^−/−^ cells for a low stretch force (800 mW). For a high stretch force (1200 mW) the depolymerization of actin through Lat A further increased the deformability to similarly high levels in both cell types. These results hence showed that the fluidization of actin seems to be the driving factor in the increased deformability of cells upon removal of Rac1.

Since we hypothesized that cellular stiffness (inverse of deformability) is a driving factor for cell migration in 3D matrix confinements, we investigated the effect of the actin depolymerization on the migratory capacity of both cell types in 3D extracellular matrix confinements. Indeed, upon inhibition of actin polymerization using Lat A, the motility of Rac1^fl/fl^ cells into 3D collagen fiber matrices was significantly reduced, whereas the motility of Rac1^−/−^ cells was not altered. However, the motility of Rac1^−/−^ cells is decreased if these cells are subjected to additional analyses, and any further increase in deformability through the addition of Lat A may not further reduce their migration capacity in 3D extracellular matrices, indicating that these remaining weakly invasive cells may use different migration modes.

In order to assess whether the removal of Rac1 causes alterations in the transmission of forces onto their 3D confined extracellular matrix, we determined the amount of fiber displacement around invaded cells in a 3D collagen fiber matrix. Indeed, the displacement of the matrix is decreased upon Rac removal, indicating a lack of force transducing structures or an impairment of the coupling between the cytoskeleton and the plasma membrane. In line with this, also the cell-cell adhesion forces of Rac1^−/−^ cells are increased compared to Rac1^fl/fl^ cells expressing Rac1. However, it has to be taken into account that the observed differences in cell-cell adhesion may be due to altered cellular deformability, since the increased deformability can impact the cell-cell height of the two cells probed with the AFM. However, since we did not observe large differences in the cell-cell height, these results suggest that both the decreased amount of force exertion (reduced fiber displacement) and the increased amount of cell-cell adhesion forces seem to contribute to a less invasive phenotype of Rac1 knockout cells.

Our findings suggest that the increased cellular deformability, increased cell-cell adhesion forces and the decreased force exertion towards the extracellular matrix environment are responsible for the impairment of the invasion of Rac1^−/−^ cells into and through dense 3D extracellular matrices. Finally, we propose that cellular mechanical properties such as cellular deformation can serve as a mechanobiological marker that represents a general parameter for determining the migratory capability of cells within 3D extracellular matrices.

### Key findings (impact on science)

- Rac1 removal increases cellular deformability
- Increased deformability of Rac1^−/−^ cells seems to be based on increased actin fluidization
- By means of depolymerization of actin, the mechanical phenotype of Rac1^fl/fl^ cells can be converted into that of Rac1^−/−^ cells
- Rac1 removal decreases the exertion of traction forces towards the 3D environment, indicating a lack of force-transducing structures or reduced connections of the cytoskeleton with the plasma membrane
- Rac1^−/−^ cells possess higher cell-cell adhesion forces that may decrease their capacity to migrate.

## Materials and Methods

### Cell culture of permanent Rac1^fl/fl^ and Rac1^−/−^ cells

The Rac1^fl/fl^ mouse embryonic fibroblast (MEFs) cells had previously been generated by immortalization of primary mouse embryonic fibroblasts prepared from embryonic day (E14.5) Rac1^fl/fl^ embryos with a SV40 large T antigen-transducing retrovirus^32^. For the isolation of clones that were homozygously deleted for the Rac1 gene, Rac1^fl/fl^ MEFs were transiently transfected with pCre-Pac encoding the Cre recombinase^81^ and then selected with 5 µg/ml puromycin for two weeks for Rac1 deficiency (Rac1^−/−^ cells). Isolated growing Rac1^−/−^ cell clones were selected, genomic DNA isolated and analyzed by genotyping^82,83^ as well as western blotting^32^. The loss of Rac1 protein was also confirmed by western blotting, employing an antibody that recognizes Rac1 and Rac3 equally well^32^. Notably, Rac3 expression is enriched in specific stages of brain development^84,85^, whereas Rac2 expression appears restricted to hematopoietic cells^86^. For subsequent experiments, the five Rac1^−/−^ cell clones were selected, based on relative comparability of growth rates, and termed KO3, KO13, KO17, KO22 and KO24^32^. To minimize potential clonal variability of studied phenotypes, all five cell clones were grown and analyzed separately, which allowed excluding that upon mixing a slightly dominantly growing clone might overgrow the rest of the clones. MEFs were maintained in Dulbecco’s modified Eagle’s medium (DMEM), 4.5 g/l glucose supplemented with 10% FCS (low endotoxin, <0.1 EU/ml, Biochrom, Berlin, Germany), 2 mM L-glutamine, 0.1 mM MEM non-essential amino acids, 1 mM sodium pyruvate and 1% 100 U/ml penicillin–streptomycin (complete medium, Gibco) (termed DMEM complete medium). Cells with app. 70% confluency were used between passages 6 and 20. A 0.125% trypsin/EDTA mixture was used for harvesting of cells (<1% dead cells). All other chemicals used were acquired from Sigma (Taufkirchen, Germany).

### Western blotting

Total extracts of respective cell lines were obtained by washing cells three times with ice cold PBS (phosphate buffered saline), addition of 4x Laemmli buffer and scraping cells. Extracts were boiled 4 minutes at 95 °C and separated by SDS-PAGE. Samples were transferred to PVDF membrane using Pierce G2 Fast Blotter (Thermo Scientific). After blocking in 10% dry milk in TBS-T, the membrane was incubated with Rac1 antibody (23A8, 1:1000, Merck Millipore) and peroxidase conjugated goat anti mouse antibody. Membrane was developed with LumiLight (Roche) using an Imager (ECL ChemoCam HR3.2, Intas). PVDF membrane was stained with Coomassie solution (0.1% Coomassie brilliant blue R-250 in 50% methanol, 7% acetic acid) afterwards and dried. Data were processed with Fiji and Photoshop CS6.

### Deformation analysis with the optical stretcher

For each optical stretcher analysis, Rac1^−/−^ cells, i.e. the five clones named KO3, KO13, KO17, KO22, KO24, and Rac1^fl/fl^ cells were cultured at 37 °C and 5% CO_2_ to a confluency of 70% in a T25 cell-culture flask. For measurements, they were detached for 4 min using a 0.125% trypsin/EDTA solution (PAA) and centrifuged at 125 g (770 rpm, 5 min). The cell pellet was dissolved in a centrifuge tube using culture medium. The cells were loaded into the microfluidic system of the optical stretcher, which delivers individual cells to the region of the instrument performing optical cell trapping. The microfluidic system was mounted onto an inverse microscope. The cells were mechanically examined with an automated optical stretcher, which detects the deformation of individual cells by image acquisition with a CCD camera (Basler, Switzerland), as previously described^42,87,88^. The device is a two-laser beam trap that captures and deforms individual cells by optical stress impacting onto the surface of the cell. The microfluidic system is linked to two coaxial optical fibers aligned orthogonally to a square glass capillary, which contains single cells. A single cell was caught in the optical trap of the two diverging laser beams^53^ and upon an increase to laser powers of either 800 mW (low stretching) or 1200 mW (high stretching), stretched along the laser axis (cell’s long axis). As a response to the stretch, the long axis of the cell exhibits a creep behavior. In more detail, after 1 s at laser powers of 100 mW (termed trap phase), the laser powers were increased to respective stretch power in a step-like manner for 2 s (termed stretch phase), and then the laser powers turned back to the trapping laser powers of 100 mW for 2 s (termed relaxation phase). After each single cell stretch event, consecutive, individual cells are delivered to the laser trapping region and treated likewise. The employed laser patterns for each cell were kept constant for all measurements and measurement temperature was 23 °C.

### Analysis of the cellular deformation

In each cell stretch experiment, numerous cells of each cell type and condition were measured to verify that the results achieved were typical for the whole cell population and reproducible, which was proven by repeating the experiments at least three times (during the last three years). We discarded any irregularly shaped cells, because they can rotate when stretched and cause ‘false’ (too small or large) deformations, all other cells were included without any further restrictions. Prior to the start of cell stretching, the microfluidic flow was adjusted. Upon cell trapping (with 100 mW laser powers), the flow was stopped in order to reduce cellular rotations. Air voids inside the capillary system and any type of cell deposit in the cell suspension were eliminated, to ensure a uniform and undisturbed pressure gradient of the flow in the microfluidic system. For avoiding or at least minimizing cell deposit, cells were harvested in the logarithmic phase of their cell growth for the optical stretching analysis.

Relative cell deformations were obtained by a custom-made edge detection algorithm at subpixel level using Matlab (MathWorks)^47,53^. Using feature tracking, the algorithm can solely correct small rotations of captured cells, whereas those with high rotations were excluded. All other cells were evaluated in terms of creep deformation J(t) = ε(t)/σ_0_, where ε(t) = [d(t)-d(0)]/d(0), which represents the relative deformation of cells on their axis parallel to laser beam axes (referred to as the long axis). The term σ_0_ describes the stress induced by the two lasers that relies linearly on two laser powers, which were either 800 mW or 1200 mW for cell stretching^47,89^. As cell deformations follow a non-Gaussian distribution, the values were given as median values. The analysis procedure for assessing the distributions of cell mechanics is under discussion at present, but the bootstrapping approach was employed to calculate a 95.45% confidence interval (2*SD) for median values of the deformation throughout the manuscript. In other words, after a bootstrapping distribution with 10000 iterations of median values, known as bootstrap resampling, it was amended by a Gaussian function in order to estimate the standard deviations of median values.

### Modulation of optical cell stretching measurements

Inhibitory drugs: For each inhibitor, measurement using 0.8 µM Lat A or 35 µM Rac1 inhibitor EHT1864 (two-five hours before the optical cell stretching measurement start and maintained in suspension buffer during the entire measurement) was carried out after two hours of cell treatment with respective drugs (unless otherwise stated) or with the solvent as vehicle controls. Each drug experiment was performed at least three times and several other inhibitor concentrations and incubation times were analyzed, such as 20 µM to 100 µM EHT1864 for two to five hours and 0.1 µM to 1.6 µM Lat A. To choose the optimal inhibitor concentration, different concentrations for all inhibitors were tested using 3D extracellular matrix invasion assays, and the most effective concentration was taken in which the cells were still viable^56,90,91^. For each condition measured with the optical cell stretcher, at least 411 cells up to 1742 cells were considered for data analysis (see below). The exact numbers of analyzed cells for each condition are mentioned in the results section.

### Measurements of 3D cell motility

We employed extracellular matrices to determine the 3D migration of Rac1^−/−^ cells (all clones) and Rac1^fl/fl^ cells. The collagen gel used at a concentration of 1.5 g/L was prepared on ice. Two different types of collagen, collagen R (4 mg/ml rat collagen type I, Serva, Heidelberg, Germany) and collagen G (4 mg/ml bovine collagen type I, Biochrom, Berlin, Germany) were mixed at a ratio of 1 to 2^72,91^, diluted in H_2_O and buffered in a 1 M phosphate buffered saline with a pH of 7.4 and a ionic strength of 0.7 (with a final concentration of 320 mM).

During preparation of the 3D matrix, inclusion of air voids was avoided. The solution was kept on ice and precooled solutions were used inhibiting the onset of polymerization. After filling as yet unpolymerized collagen matrices into wells, gels were polymerized at 95% humidity under 5% CO_2_ and at 37 °C, for approximately two hours. The fully polymerized gels were washed twice with PBS, and incubated overnight with DMEM complete growth medium^43,44,54^. In addition, we prepared 3 g/L collagen fiber matrices with the mixture of collagen R and collagen G in a 1 to 2 ratio, respectively.

80% confluent Rac1^−/−^ and Rac1^fl/fl^ fibroblast cells were harvested. 50.000 cells were allowed to spread on and invade into the surface of 3D extracellular matrices and cultured for three days at 37 °C with 95% humidity and 5% CO_2_. After three days, the invasiveness of the two fibroblast cell types was clearly different and hence defined as optimal time point for consecutive measurements. More precisely, the positions of non-invasive cells determined by their nuclei served as markers for the outermost layer of the gel, which represents the topmost collagen fibers. After three days, cells were fixed with a 2.5% glutaraldehyde solution (Serva, Heidelberg, Germany) and stained with Hoechst 33342 (10 µg/ml in PBS). Thereafter, cells can be stored in 1X PBS at 4–5 °C for imaging. In more detail, the percentage of invasive cells and their invasion depths were ascertained in at least 100 randomly selected fields of view located in the center of each six well. Z-stacks harboring at least 100 images were recorded at 4 μm steps using a 20x objective and a CCD Orca camera (Hamamatsu Photonics, Hersching am Ammersee, Germany), mounted on a 0.55x c-mount. Cell nuclei were detected using a customized 3D cell nucleus detection algorithm written in Python. A cell is identified as invasive when the nucleus is found to be located below the topmost cell-layer of non-invasive cells. Due to the depth of field for the 20 × 0.4 NA objective, the uncertainty of this method is approximately 5 µm. To determine the proportion of invasive cells, non-motile cells adhering to the surface of the collagen gel were enumerated.

For the cell cluster analysis, we used the DBSCAN algorithm^92^. The DBSCAN clustering algorithm can detect individual cells and cells in a cluster seperately^93^. Cells are considered to belong to a cluster if such a cluster contains at least 5 cells and their nuclei lie within a distance of 10 µm.

### Interference with cell invasion using pharmacological inhibitors

To modulate or abolish cellular invasiveness, we added 35 µM Rac1 inhibitor EHT1864 and 0.8 µM Lat A to the collagen invasion assay twelve hours after cell seeding. The total duration of the invasion assay was three days. Then, the cells were treated and analyzed as described above.

### Immunofluorescence using a confocal laser scanning microscope on flat substrates

For 2D-coated glass cover slides and cell stimulation with inhibitory drugs: For proper cell adhesion, the cleaned glass slides were coated with different extracellular matrix proteins such as 10 µg/ml laminin for two hours at 37 °C. For removal of unbound extracellular matrix protein, the coated slides were washed twice with PBS buffer. 4000 to 8000 cells were seeded and cultured for 16 hours either in the absence (vehicle control: DMSO buffer, if not indicated otherwise) or presence of 35 µM Rac1 inhibitor (EHT1864) (vehicle control: water) or 0.8 µM Lat A for two hours at 37 °C. For termination of cell treatments, cells were washed with PBS buffer and fixed with 4% paraformaldehyde for 10 min at room temperature. After washing fixed cells with PBS buffer at least twice, cells were blocked with 1% bovine serum albumin (BSA) in PBS buffer for 20 min to reduce background noise of the fluorescent staining. In more detail, the cells were treated with 5 units/ml Alexa Fluor 546 Phalloidin (in 1% BSA HEPES-buffer), 0.25 mg/ml DID and 0.02 mg/ml Hoechst 33342 for 4 hours at room temperature. For the reduction of photobleaching, glass cover slips were treated with prolong gold antifade (No. 369394, Life Technologies, Eugene, Oregon, USA) before placing them onto a glass slide. For obtaining optimal results, coverslips on embedding medium containing antifade were stored for 24 hours at 4 °C until a gel-like consistency was achieved, followed by their sealing with nail polish and analysis under a confocal laser scanning microscope (TCS SP8, Leica, Wetzlar, Germany).

### Imaging of cells upon invasion into 3D extracellular matrices using confocal laser scanning microscopy

Before preparation of 3D matrices, glass coverslips were incubated at 120 °C for two hours, activated with a plasma-cleaner for five minutes, silanized with (3-aminopropyl) trimethyoxysilane using a spin-coater (Sigma Aldrich), washed 2–3 times with double-distilled endotoxin-free water (dH_2_O), incubated with 2.5% glutaraldehyde at 37 °C and washed again. Subsequently, cleaned glass coverslips were coated with a non-polymerized collagen type I gel solution (see 3D motility assays above) for two hours at 37 °C, washed 2–3 times with dH_2_O and soaked with culture medium for one day. Between 3000 and 5000 cells were plated onto the collagen matrices placed on top of coverslips (for gel preparation see invasion assays) and cultured for two days. Subsequently, fixation was performed using 2.5% glutaraldehyde, followed by washing twice with PBS-buffer, permeabilization with 0.1% Triton-X100 (5 min at room temperature), repeated washing and overnight storage in 1% BSA Hepes-buffer. For analysis of the actin cytoskeleton, cells were incubated with 10 units/ml Alexa Fluor 546 (1% BSA Hepes-buffer) at 4 °C for 2.5 hours. After rinsing with 1% BSA, samples were incubated with prolong gold antifade. After one day storage at 4 °C, the antifade behaved like a gel, ready for sealing and imaging by confocal laser scanning microscopy. The actin cytoskeleton of cells formed in 3D extracellular matrices was explored by taking stacks of images.

### Atomic force microscopy

We used atomic force microscopy (AFM) to determine the cell-cell adhesion forces between two cells of the same cell type, i.e. either Rac1^−/−^ cells (clone KO17) or Rac1^fl/fl^ cells as controls. The tipless cantilevers (Arrow-TL2-50, force constant 0.03 N/m, Nanoworld, Neuchatel, Switzerland) were first incubated overnight at 4 °C with 20 µg/ml fibronectin. Before each measurement, the glass block of the AFM was treated with 70% ethanol and dH_2_O. The spring constants of cantilevers were analyzed in dH_2_O using the thermal noise approach. In particular, the second peak of the power spectrum was chosen for the fit, since the first peak is not suitable when the AFM measurement is performed in a medium containing mainly water^70,71^. For measurements, 5000 to 6000 cells were seeded into plastic culture dishes (3.5 cm in diameter) and placed onto the microscopic stage (heated to 37 °C and maintained in a 5% CO_2_ atmosphere. The fibronectin-coated, tipless cantilever was then directly after cell seeding placed into the suspension of cells to attach the first cell to the cantilever. In the next step, the cantilever carrying a cell and the adherent cells in a culture dish are incubated for at least two hours before measurement start of cell-cell adhesion forces. In more detail, the adherent cells were analyzed by applying the cell-carrying cantilever with a force of 0.5 nN on top of another cell attached to the substrate for 5 s to 100 s, as indicated. Curves for cell-cell approach and retraction force-distance were acquired. The maximal force for each cell-cell adhesive interaction was obtained using JPK data processing software, where offset and tilt were corrected, and subsequently the minimum of the retraction curve determined, which represents the maximal adhesion force between the two cells (one attached at the cantilever and one adhered to the culture dish surface).

### Plate rheology measurements

After the addition of buffer solution and water (tube 1), both type I collagen types, i.e. R (rat tail, 4 g/L) and G (bovine skin, 4 g/L) were mixed (tube 2). Both tubes were stored on ice. Before measurement start, both tube contents were mixed, and 200 µl of this mix was pipetted underneath a rounded plate with a diameter of 25 mm and a distance of 400 µm to the plate. The lid was closed and the whole gel was incubated for 15 min at 37 °C (the incubation systems included heating, water bath and incubation chamber). As a blocking buffer, we used 2250 µl of a collagen buffer system (instead of collagen, H_2_O is used). The fibrillation phase has a duration of two hours at 37 °C and has not been measured. After fibrillation using a dynamic shear rheometer (ARES, TA Instruments, USA), the measurement was performed with frequencies ranging from 0.01 to 20 Hz, and a strain γ of 2% and 21 data points per decade were analyzed.

### Fiber displacement measurement in 3D extracellular matrix confinements

Rac1^−/−^ and Rac1^fl/fl^ control cells were seeded onto 3D collagen matrices that had previously been polymerized in 24 well μPlates (Ibidi, Cat. No. 82406). The seeded cells were incubated for 24 hours in a cell incubator at 5% CO_2_, 95% humidity and 37 °C. Prior to any measurement, the cell membrane was fluorescently labeled using Vybrant DiD Cell-Labeling Solution (Thermofischer, Cat. No. V22887). Probes with randomly chosen individual cells (n = 13 cells for Rac1^fl/fl^ cells and n = 14 cells for Rac1^−/−^ KO17cells) that migrated inside the 3D scaffold were placed under a Confocal Laser Scanning Microscope with incubation chamber (TCS SP8, Leica). The Confocal Laser Scanning Microscope enabled live imaging of both color channels for transmitted light (the matrix) and Vybrant DiD (the cell). 3D images were recorded every 3–5 minutes over a total time period of 8–24 hours. Commonly, the first two hours were included in this manuscript. Cell motility did not affect the analysis, as the fiber displacements were calculated between each consecutive time frame. However, it may occur that cells migrate toward the edge or even out of the recorded imaging volume. In this case, the recording was cut accordingly, to assure a proper analysis result.

The scaffold deformation has been related to the amount of pulling force the cell exerts on the 3D collagen fiber matrix, similar to traction force measurements.

Firstly, the displacement of the fibers within the extracellular matrix confinement is analyzed quantitatively by applying the well-known LucasKanadeAlgorithm^50,94^ implemented in 3D in a custom-written Python program to the matrix channel of the laser scanning microscopy images. Small displacements caused by background drifts were compensated by subtracting the zero frequency of a FastFourierTransform analysis. This resulted in a 4D vector field of 3D fiber displacements over time. The absolute fiber displacement was calculated from the vector field for each image pixel and consecutive time point, which represents the general, direction-independent displacement of the collagen fibers. Secondly, the 3D cell shape was segmented using the membrane channel from the laser scanning microscopic images, yielding the cell boundary. Thirdly, the median fiber displacement in consecutively outgoing 3D shells with a thickness of about 2 µm around the cell boundary were calculated for 20 such shells (see Fig. 3E). Finally, these fiber displacement values were used to quantify the amount of fiber displacement around individual invasive cells in dependence of the distance from the cell boundary and over time. Exemplary vector fields of the fiber displacements of both cell types are shown in Fig. 3H,I, in which larger arrows represent larger displacement.

### Statistical assessment

The data were provided as mean or median values ± SD, as indicated. For statistical analysis, the Wilcoxon-Mann-Whitney U-test (optical cell stretching), Welch’s t-test (invasion data) and independent t-test (cell-cell adhesion and indentation, matrix fiber displacement) were carried out to discern statistically significant differences of the data when appropriate.

## Supplementary information

Supplementary material

## Data Availability

The datasets generated during and/or analyzed during the current study are available from the corresponding author on reasonable request.
