# Supplementary material for: The Small GTPase Rac1 Increases Cell Surface Stiffness and Enhances 3D Migration Into Extracellular Matrices
Source: Sci Rep. 2019 May 22;9:7675. doi: 10.1038/s41598-019-43975-0 (PMC6531482; doi:10.1038/s41598-019-43975-0)
Supplement: Supplementary file 1 — Supplementary material [file 41598_2019_43975_MOESM1_ESM.pdf]

Supplemental material

The small GTPase RAC1 increases cell surface stiffness and enhances 3D migration into extracellular matrices

Tom Kunschmann, Stefanie Puder, Tony Fischer, Anika Steffen, Klemens Rottner and Claudia Tanja Mierke

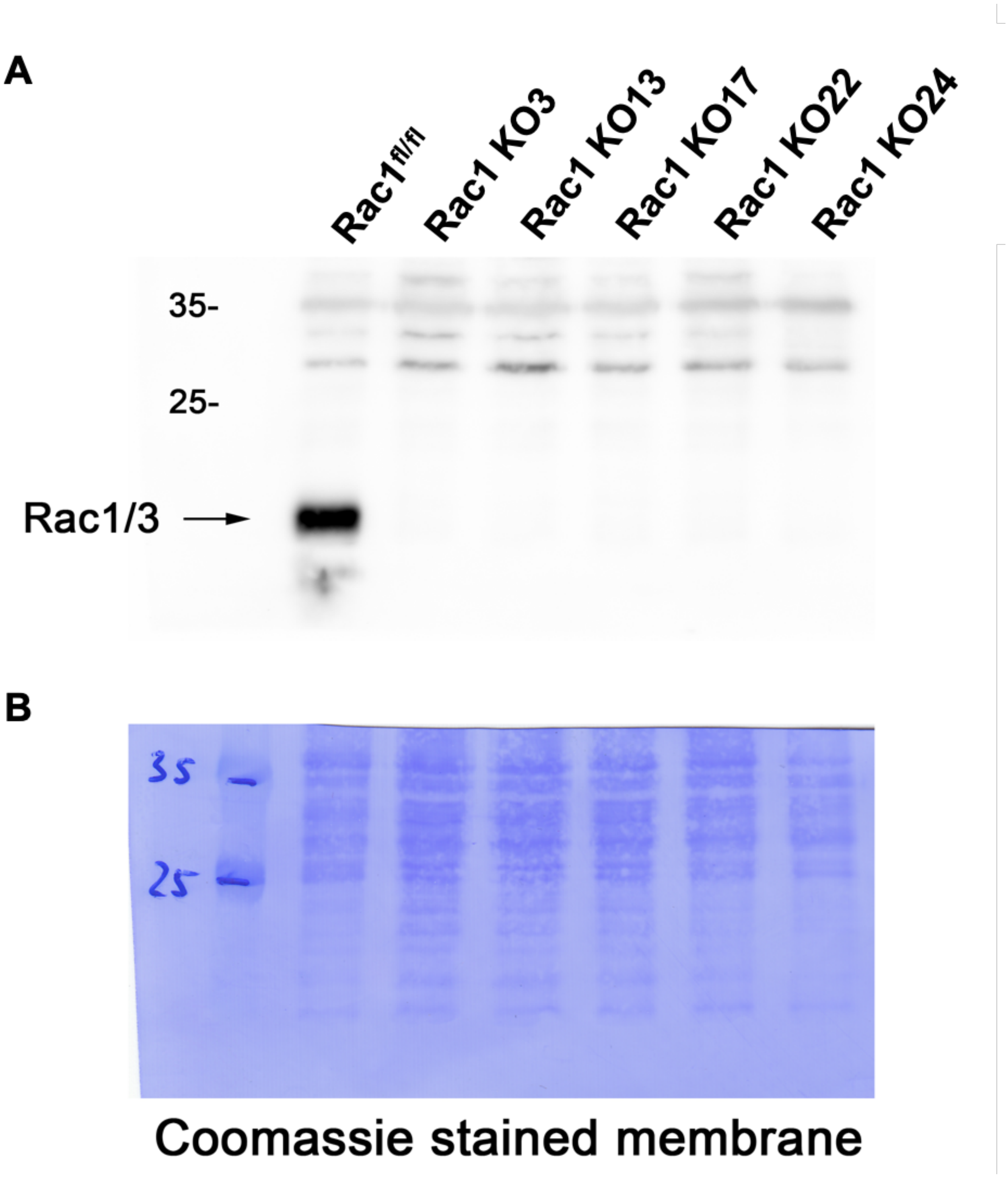

Figure S1: Western blot of cell lines employed. A) Whole cell extracts were developed with an antibody to Rac1/3. Note the complete absence of Rac1 and Rac3 in all KO cell lines. B) The membrane of the western blot was stained with Coomassie afterwards to reveal protein load.

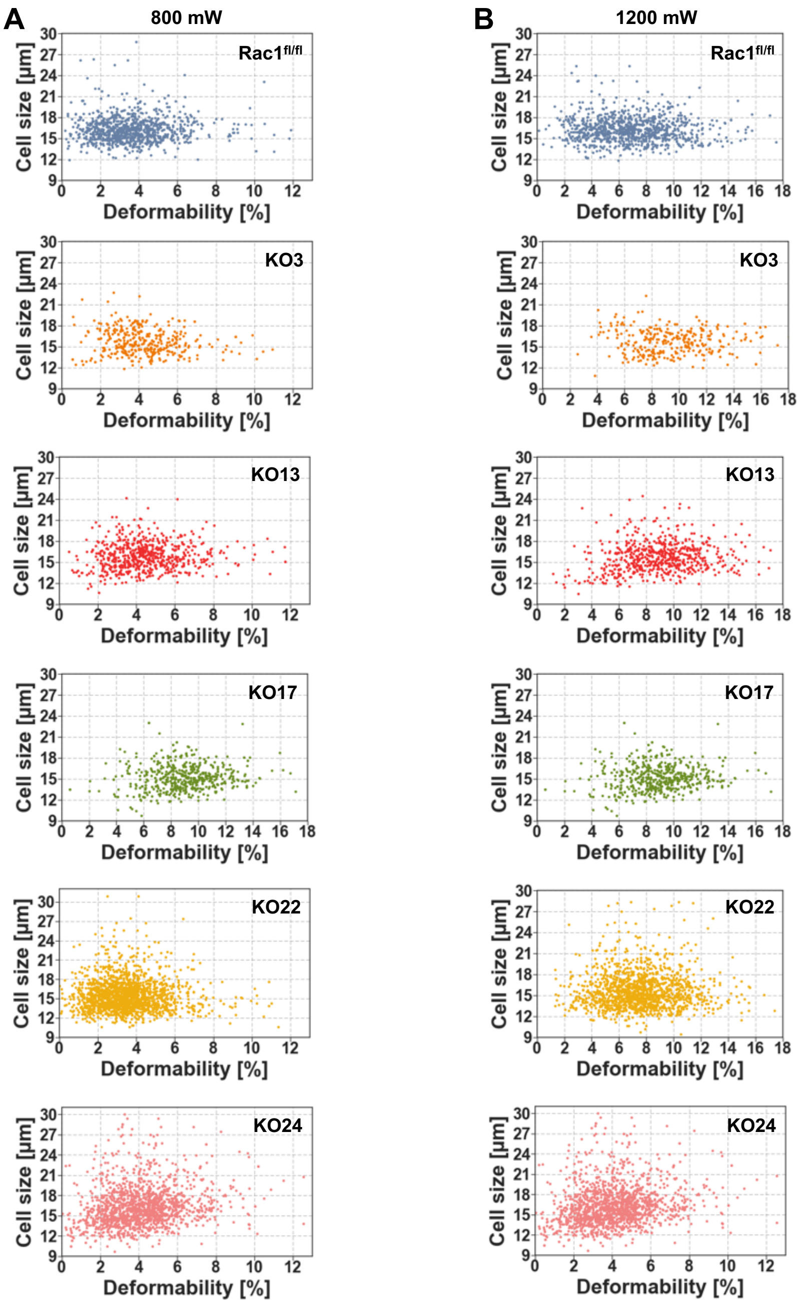

Figure S2: The relative deformation of the six cell types (Rac1$^{fl/fl}$ and the five Rac1$^{-/-}$ cell clones such as KO3, KO13, KO17, KO22 and KO24) is plotted over cell size for both laser stretching powers. The relative deformation is not correlated with the cell size and the cell sizes are comparable between all six cell types.

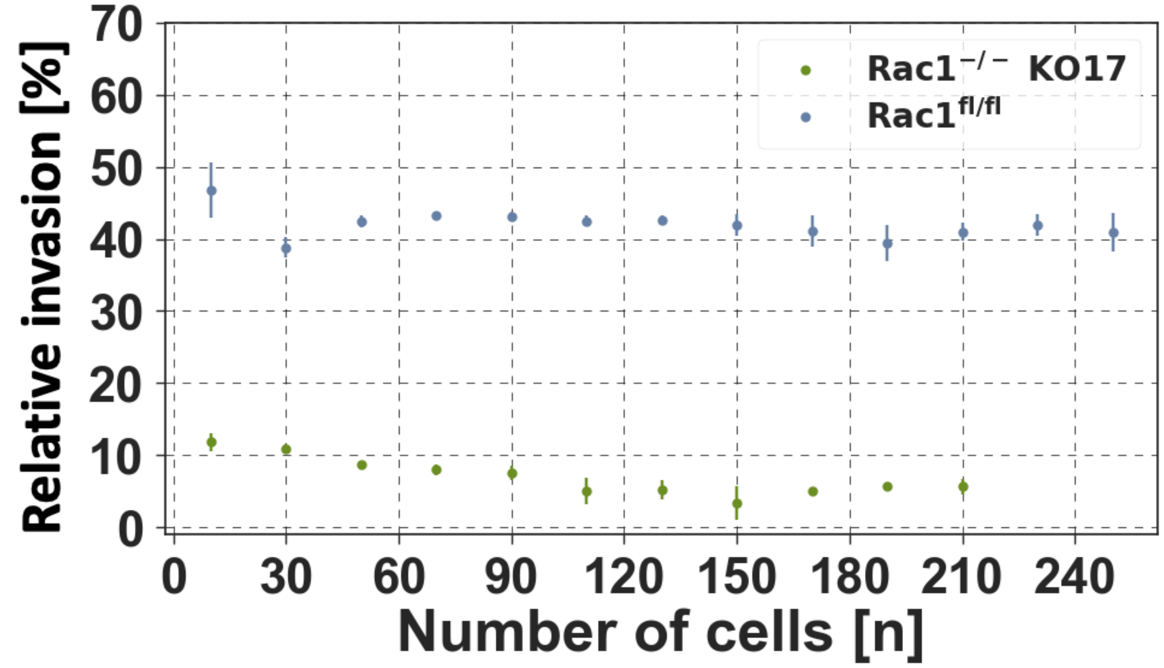

Figure S3: The percentage of invasive Rac1$^{fl/fl}$ cells (blue) and Rac1$^{-/-}$ cells (green, clone KO17) is not altered by the number of cells.

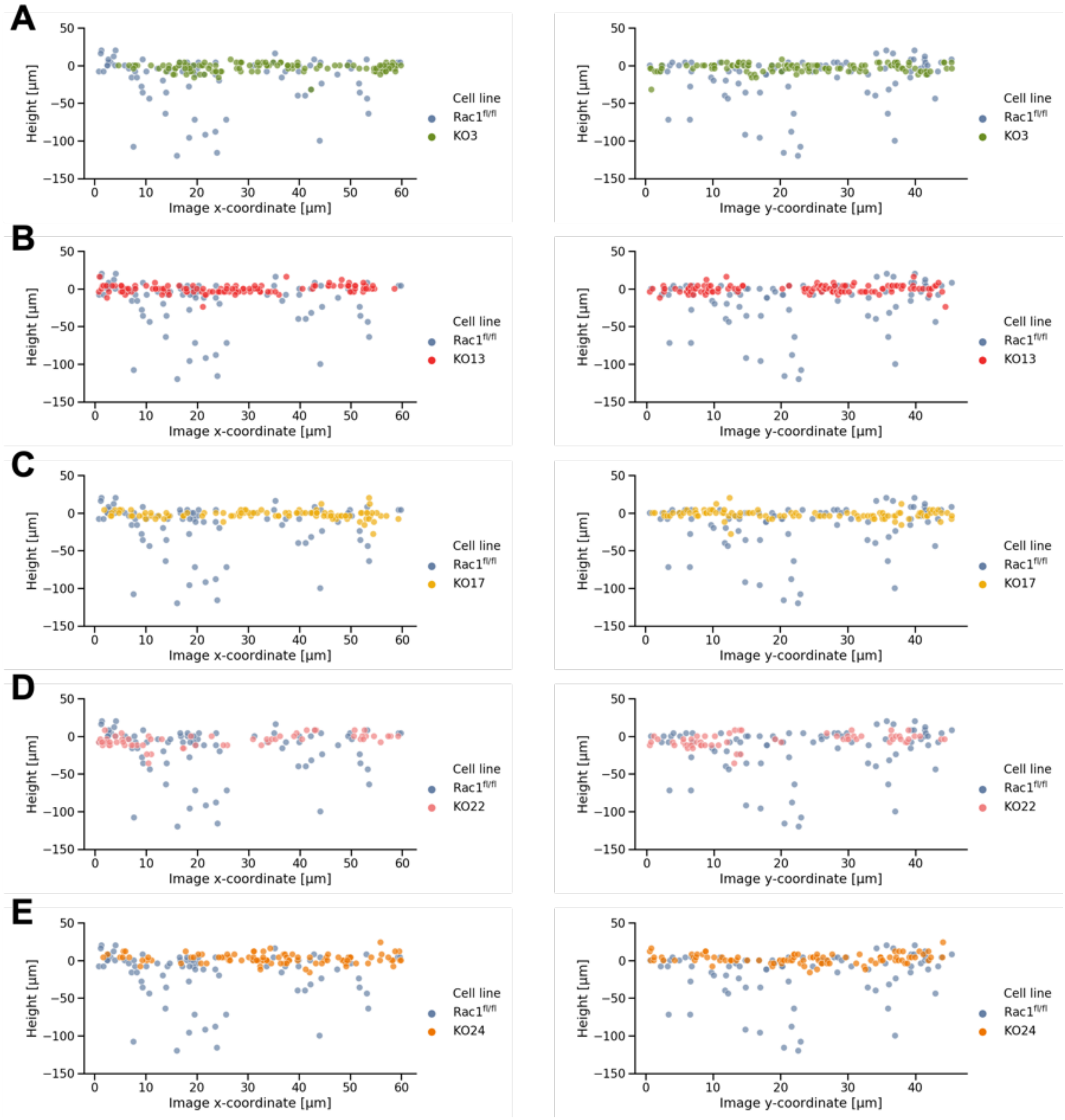

Figure S4: Representative raw data showing cell migration inside the 3D collagen fiber matrices are provided for all five Rac1$^{-/-}$ cell clones, such as KO3 (A), KO13 (B), KO17 (C), KO22 (D) and KO24 (E), and they are all compared to the Rac1$^{fl/fl}$ control cells (blue).

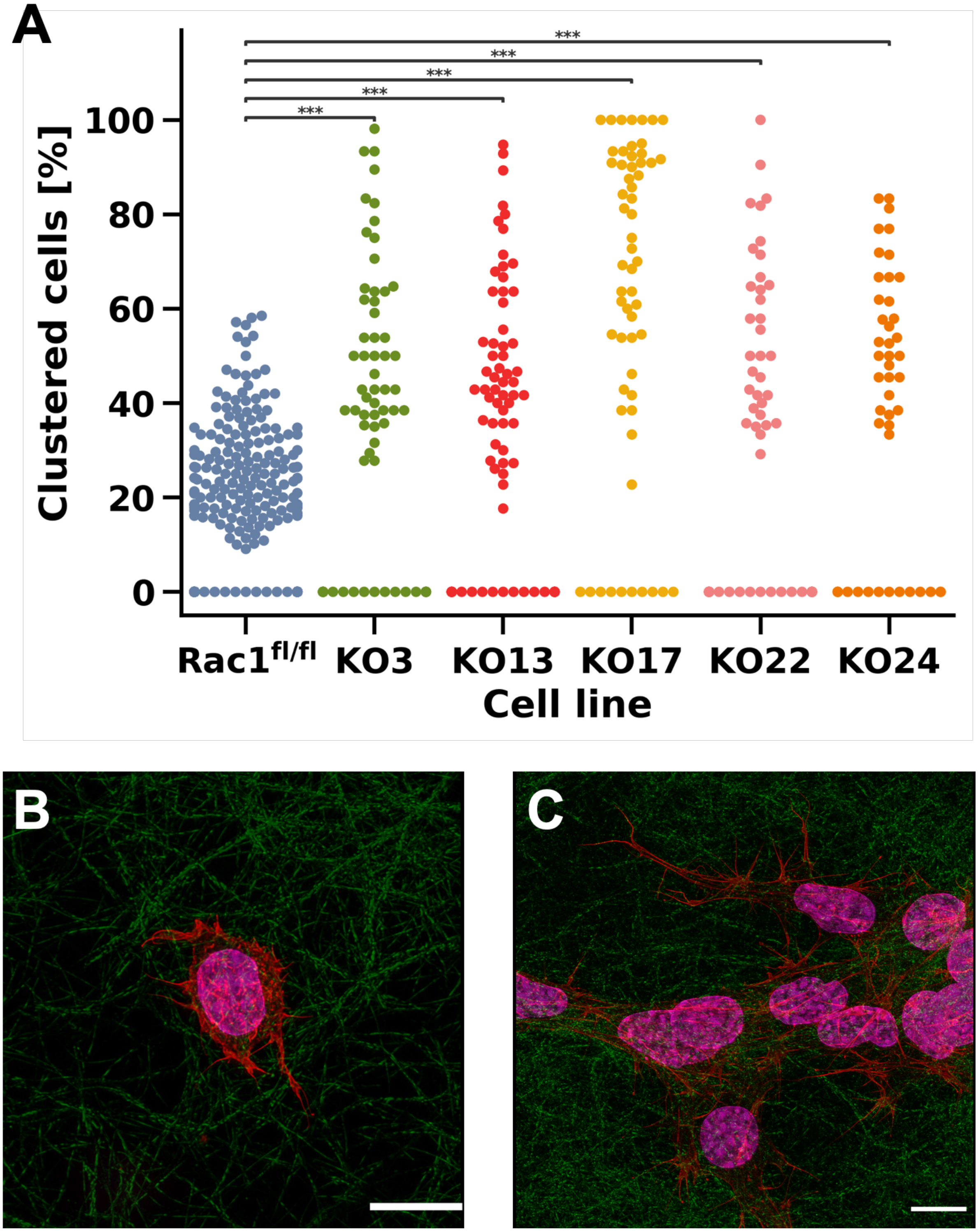

Figure S5: Single cell and collective migration of Rac1$^{-/-}$ cell clones, such as KO3, KO13, KO17, KO 22 and KO24, and Rac1$^{fl/fl}$ cells. A) The percentage of clustered cells among invasive cells after three days of invasion into 1.5 mg/ml 3D collagen matrices. ***p< 0.001. Laser scanning confocal images of representative images of a single cell (Rac1$^{fl/fl}$ cell, B) and a collection of cells (Rac1$^{-/-}$ cell, KO17) that migrated into a 1.5 mg/ml collagen fiber matrix after three days. The actin filaments are fluorescently labelled using Alexa Fluor 546 Phalloidin (red), staining collagen fibers are visualized by Second harmonic generation (SHG, green), and the nuclei are stained with 33342 Hoechst (pink). Scale bars are 10 µm.

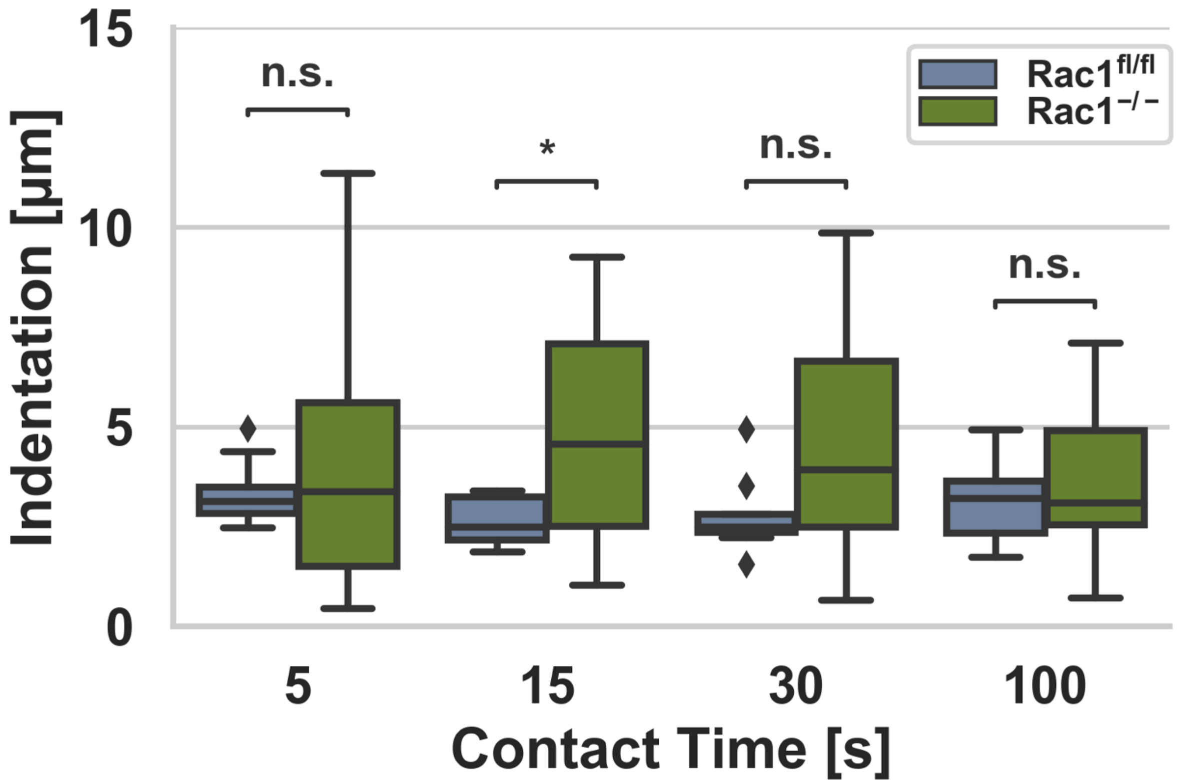

Figure S6: The indentation of the cantilever-attached cell towards the substrate-adhered cell is plotted over the cell-cell contact time between Rac1$^{fl/fl}$ cells (blue) and Rac1$^{-/-}$ cells (green, clone KO17). *$p < 0.05$, n.s. = not significant
